# Evidence for a Contribution of ALA Synthesis to Plastid-To-Nucleus Signaling

**DOI:** 10.3389/fpls.2012.00236

**Published:** 2012-10-29

**Authors:** Olaf Czarnecki, Christine Gläßer, Jin-Gui Chen, Klaus F. X. Mayer, Bernhard Grimm

**Affiliations:** ^1^Department of Plant Physiology, Institute of Biology, Humboldt-Universität zu BerlinBerlin, Germany; ^2^Plant Systems Biology, Biosciences Division, Oak Ridge National LaboratoryOak Ridge, TN, USA; ^3^Institute of Bioinformatics and Systems Biology, German Research Center for Environmental Health, Helmholtz Zentrum MünchenNeuherberg, Germany

**Keywords:** ALA synthesis, retrograde signaling, gabaculine, *gun* mutants, microarray analysis

## Abstract

The formation of 5-aminolevulinic acid (ALA) in tetrapyrrole biosynthesis is widely controlled by environmental and metabolic feedback cues that determine the influx into the entire metabolic path. Because of its central role as the rate-limiting step, we hypothesized a potential role of ALA biosynthesis in tetrapyrrole-mediated retrograde signaling and exploited the direct impact of ALA biosynthesis on nuclear gene expression (NGE) by using two different approaches. Firstly, the *Arabidopsis* *gun1*, *hy1* (*gun2*), *hy2* (*gun3*), *gun4* mutants showing uncoupled NGE from the physiological state of chloroplasts were thoroughly examined for regulatory modifications of ALA synthesis and transcriptional control in the nucleus. We found that reduced ALA-synthesizing capacity is common to analyzed *gun* mutants. Inhibition of ALA synthesis by gabaculine (GAB) that inactivates glutamate-1-semialdehyde aminotransferase and ALA feeding of wild-type and mutant seedlings corroborate the expression data of *gun* mutants. Transcript level of photosynthetic marker genes were enhanced in norflurazon (NF)-treated seedlings upon additional GAB treatment, while enhanced ALA amounts diminish these RNA levels in NF-treated wild-type in comparison to the solely NF-treated seedlings. Secondly, the impact of posttranslationally down-regulated ALA synthesis on NGE was investigated by global transcriptome analysis of GAB-treated *Arabidopsis* seedlings and the *gun4-1* mutant, which is also characterized by reduced ALA formation. A common set of significantly modulated genes was identified indicating ALA synthesis as a potential signal emitter. The over-represented gene ontology categories of genes with decreased or increased transcript abundance highlight a few biological processes and cellular functions, which are remarkably affected in response to plastid-localized ALA biosynthesis. These results support the hypothesis that ALA biosynthesis correlates with retrograde signaling-mediated control of NGE.

## Introduction

Organellar genes translocated to the nucleus of host organisms during the process of evolution remain necessary for the function of organelles that now support important processes within their hosts. For example, several thousand nucleus-encoded proteins are required for chloroplast biogenesis, including the formation of complexes consisting of both nuclear and plastid-encoded subunits (Timmis et al., 2004). This bigenome-dependent assembly of functional protein complexes requires a finely tuned balance of anterograde and retrograde control. While anterograde control determines organellar metabolism and gene expression, nuclear gene expression (NGE) are under retrograde control responding to organelles. However, detailed knowledge about signaling molecules and pathways is still lacking (Papenbrock and Grimm, 2001; Brusslan and Peterson, 2002; Surpin et al., 2002; Gray et al., 2003; Pfannschmidt et al., 2003; Baier and Dietz, 2005; Beck and Grimm, 2006; Pogson et al., 2008; Kleine et al., 2009; Jung and Chory, 2010).

**Figure 1:**
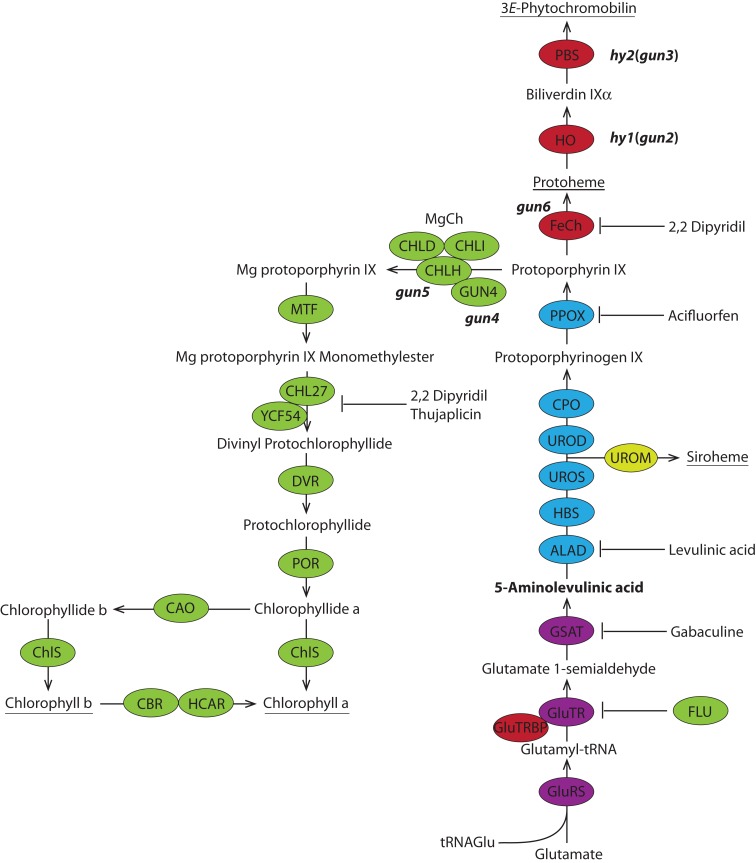
**Metabolic pathway of tetrapyrrole biosynthesis in plants**. The color code comprise the five sections of the tetrapyrrole biosynthetic pathway: ALA synthesis purple, porphyrin synthesis blue, the Fe branch red, Mg branch green, siroheme synthesis yellow. The universal precursor of all tetrapyrroles 5-aminolevulinic acid (ALA) is synthesized from glutamate via a three-step reaction. ALA is further metabolized to protoporphyrin IX before the pathway branches into heme (and phytochromobilin) and chlorophyll biosynthesis. The ratio of chlorophyll a and b is balanced in the chlorophyll cycle. The end products of the pathway are underlined. Mutants of tetrapyrrole biosynthetic enzymes that play a role in plastid-to-nucleus signaling and inhibitors of certain enzymatic steps are shown. ALAD, ALA dehydratase; CAO, Chl a oxygenase; CBR, chlorophyll b reductase; ChlS, chlorophyll synthase; CPO, coproporphyrinogen III oxidase; DVR, divinyl protochlorophyllide reductase; FeCh, Fe chelatase; FLU, flourescent; GluRS, glutamyl-tRNA synthetase; GluTR, glutamyl-tRNA reductase; GluTRBP, GluTR binding protein; GSAT, glutamate-1-semialdehyde aminotransferase; HBS, hydroxymethylbilane synthase; HCAR, 7-hydroxymethyl chlorophyll a reductase; HO, heme oxygenase; MgCh, Mg chelatase; MTF, Mg protoporphyrin IX methyltransferase; PBS, phytochromobilin synthase; POR, light dependent NADPH-protochlorophyllide oxidoreductase; PPOX, protoporphyrinogen IX oxidase; UROD, uroporphyrinogen III decarboxylase; UROM, uroporphyrinogen III methyltransferase; UROS, uroporphyrinogen III synthase.

Physiological and genetic analyses of NGE modulation indicate that tetrapyrrole-mediated signaling may be an important mechanism for retrograde control over NGE. Down-regulation of individual enzymatic steps of tetrapyrrole biosynthesis (Figure 1), as a result of either gene silencing or enzyme inhibitors, has been shown to reduce the expression of a *LHCB* gene encoding a light-harvesting chlorophyll (Chl)-binding protein of photosystem II in a number of studies. For example, in initial experiments, the iron-chelating 2,2-dipyridyl was applied to light-exposed cultures of the green algae *Chlamydomonas reinhardtii*, resulting in accumulation of protoporphyrin IX (Proto) and Mg protoporphyrin IX monomethylester (MgProtoME) and, concomitantly, in reduced *LHCB* mRNA content (Johanningmeier and Howell, 1984). Later studies found increased levels of *LHCB* transcripts in dark-grown *C. reinhardtii* cultures fed with Mg porphyrin, as well as in *Chlamydomonas* *CHLH* and *CHLD* (Figure 1) deletion mutant strains where Mg porphyrin had accumulated, which underlines the correlation between Mg porphyrin-mediated signaling and NGE (Kropat et al., 1997; La Rocca et al., 2001; Vasileuskaya et al., 2004). The accumulation of MgProtoME in dark-incubated cress seedlings through inhibition by thujaplicin resulted in elevated *LHCB* mRNA content that was likely related to a metabolic response resembling light-induced control of nuclear genes for photosynthesis and plastid development (Oster et al., 1996). Last, inactivation of Mg chelatase, Mg protoporphyrin IX (MgProto) methyltransferase, and Chl synthase (Figure 1) resulted in altered expression of multiple nuclear-encoded genes in the tetrapyrrole biosynthesis in transgenic tobacco plants (Papenbrock et al., 2000b; Alawady and Grimm, 2005; Shalygo et al., 2009).

A mutant screen of *Arabidopsis* seedlings treated with norflurazon (NF), an inhibitor of carotenoid biosynthesis, identified components of retrograde signaling contributing to un-coupling of *LHCB* expression from chloroplast development (Susek et al., 1993). Four out of five *genomes uncoupled* (*gun*) mutants were reported to encode proteins involved in tetrapyrrole biosynthesis (Figure 1). *GUN2* and *GUN3*, encoding the heme oxygenase and the phytochromobilin synthase (Mochizuki et al., 2001), are allelic to *hy1* and *hy2*, respectively. The *Arabidopsis* *hy* mutants have been initially identified in mutant screens for deficits in light signaling (Chory et al., 1989). These mutants are phytochrome-deficient due to impaired synthesis of the chromophore phytochromobiline. *GUN4* encodes a regulator of Mg chelatase activity (Larkin et al., 2003; Peter and Grimm, 2009) and *GUN5* encodes the CHLH subunit of Mg chelatase (Mochizuki et al., 2001). *GUN1* encodes a plastid-localized protein of the pentatricopeptide-repeat protein family that acts independently of tetrapyrrole biosynthesis (Koussevitzky et al., 2007). Accumulation of MgProto was compared from wild-type, *gun5* and *gun2* and it has been proposed that due to its prevalence MgProto likely acts as a signaling molecule for the expression of *LHCB1* and other nuclear-encoded genes (Strand et al., 2003). However, in more recent studies, no clear correlation was shown between steady-state levels of MgProto and *LHCB* expression in NF-treated *Arabidopsis* seedlings. It was suggested that phenotypical differences between *gun* and wild-type seedlings, including *LHCB* expression, may be attributed to ROS-based retrograde signaling (Mochizuki et al., 2008; Moulin et al., 2008). In addition, transgenic plants overexpressing *FCI* encoding ferrochelatase I (Figure 1) show *gun*-like altered expression of photosynthesis-associated nuclear genes (PhANGs) highlighting heme as a potential signaling molecule in plastid-to-nucleus communication (Woodson et al., 2011).

Taken together, this evidence indicates that tetrapyrrole intermediates, such as Mg porphyrins, and endproducts, such as heme, are potential emitters of plastid-derived retrograde signals generated from tetrapyrrole metabolism. However, steady-state levels of tetrapyrrole metabolites and endproducts are also usually correlated with the synthesis of 5-aminolevulinic acid (ALA, Figure 1), which is the rate-limiting step of tetrapyrrole biosynthesis (Papenbrock et al., 2000a; Alawady and Grimm, 2005; Shalygo et al., 2009; Peter et al., 2010).

In cyanobacteria, algae and plants ALA is synthesized in a three-step reaction including activation of glutamate by binding to a plastid tRNA^Glu^ followed by a reduction of glutamate to glutamate-1-semialdehyde catalyzed by glutamyl-tRNA reductase (GluTR) and a final transamination mediated by glutamate-1-semialdehyde aminotransferase (GSAT; Figure 1). Following synthesis, ALA is further processed in the branched pathway leading to the tetrapyrrole end products Chl, heme, siroheme, and phytochromobilin (Figure 1; Papenbrock and Grimm, 2001; Tanaka and Tanaka, 2007).

5-Aminolevulinic acid synthesis is controlled at the transcriptional and posttranslational level by changes in environment (e.g., light, temperature) and endogenous stimuli, such as hormonal and metabolic feedback regulation. Furthermore, several feedback-control mechanisms affect ALA biosynthesis on a posttranslational level. The negative regulator FLU binds to GluTR and mediates the suppression of ALA synthesis in the dark (Meskauskiene et al., 2001; Meskauskiene and Apel, 2002), most likely by interaction with protochlorophyllide oxidoreductase (POR) and in response to immediate accumulating Pchlide (Richter et al., 2010; Kauss et al., 2012). For example, the *Arabidopsis* *flu* mutant does not down-regulate GluTR activity, and accumulates Pchlide in darkness which subsequently generates singlet oxygen upon light exposure (Op den Camp et al., 2003).

Our aim was to examine the strong regulatory impact of ALA synthesis on tetrapyrrole biosynthesis, including its potential role in retrograde signal-mediated NGE derived from tetrapyrrole metabolism. We show that reduced ALA synthesis in *gun4-1* mutants, and inhibition of ALA synthesis by gabaculine (GAB) in wild-type *Arabidopsis* seedlings, lead to elevated transcript levels of photosynthetic genes if treated with NF. Taken together, these data support the idea that ALA-mediated retrograde signaling may modify NGE in *Arabidopsis* plants.

## Materials and Methods

### Plant growth conditions

The *hy1* and *hy2* mutants of *Arabidopsis thaliana* L. originated from the ecotype Landsberg erecta (Ler-0) background (Chory et al., 1989), and *gun1-1* and *gun4-1* mutants (Col-0 background) were initially described in Susek et al. (1993) and Larkin et al. (2003), respectively. For analysis of etiolated, de-etiolated, and 7-day-old wild-type and mutant seedlings, seeds were surface-sterilized and plated onto 0.5× MS medium (Murashige and Skoog, 1962) supplemented with 1% (w/v) sucrose 0.8% (w/v) agar. After vernalization at 4°C for 2 days, plates and pots were transferred to a growth chamber with light intensity of 100–120 μmol photons m^−2^ s^−1^ at 22–23°C under a 12-h light/12-h dark regime. Etiolated seedlings were exposed to light for 2 h to synchronize germination before they were dark-incubated for 3 days. Ten micromolars of GAB (5-aminocyclohexa-1,3-diene-1-carboxylic acid) or 100 μM ALA or 1 μM NF were supplied to the MS medium as indicated in the results.

### Determination of ALA

5-Aminolevulinic acid content was measured using the method from Mauzerall and Granick (1956). Seedlings or leaves (100–200 mg fresh weight) were harvested 1 h after the start of illumination and incubated in a buffer containing 50 mM Tris-HCl (pH 7.2) and 40 mM levulinate for 3 h at 22–23°C and 100 μmol photons m^−2^ s^−1^. Following the incubation, plant material was dried, weighed, and frozen in liquid nitrogen. Frozen samples were homogenized, resuspended in 20 mM K-phosphate buffer (pH 6.8) and centrifuged for 10 min at 16,000 × *g*. Four hundred microliters of the supernatant were mixed with 100 μl ethyl acetoacetate and boiled for 10 min at 100°C. Samples were mixed with 500 μl modified Ehrlich’s reagent (373 ml acetic acid, 90 ml 70% v/v perchloric acid, 1.55 g HgCl_2_, 9.10 g 4-dimethylaminobenzaldehyde diluted to 500 ml with H_2_O) and centrifuged for 5 min at 16,000 × *g*. Absorption was measured at 526, 553, and 720 nm and the ALA content of the samples was calculated using a standard curve generated by commercial ALA (Sigma–Aldrich Inc.).

### Determination of the Pchlide content

Extraction of Pchlide was performed according to the protocol of Koski and Smith (1948). Etiolated *Arabidopsis* WT and mutant seedlings (100–300 mg fresh weight) were treated with steam for 2 min, ground in liquid nitrogen, and extracted three times in alkaline acetone (9 vol 100% acetone: 1 vol 0.1 N NH_4_OH). After centrifugation at 16,000 × *g* for 10 min the supernatants were collected and Pchlide content was determined by fluorescence spectroscopy. Extracts were excited at 433 nm and fluorescence emission was recorded at 632–633 nm. For calibration, a Pchlide standard was extracted from 7-day-old etiolated barley leaves (Koski and Smith, 1948) and quantified using the Pchlide extinction coefficient in diethyl ether at 623 nm of 35,600 M^−1^ cm^−1^ (Dawson et al., 1986).

### Pigment analysis

Chlorophyll and carotenoids were extracted from 100 mg (fresh weight) of seedlings or leaf material with 80% (v/v) acetone containing 10 μM potassium hydroxide. Supernatants of three extraction steps were combined and analyzed spectrophotometrically (Lichtenthaler, 1987; Czarnecki et al., 2011b).

### Quantitative real-time PCR

Total RNA of seedlings was extracted using the Invisorb Spin Plant RNA Mini Kit (Invitek, Germany). A cDNA was reversely transcribed using 2 μg total RNA, oligo-dT_18_ Primer and RevertAid™ M-MuLV Reverse Transcriptase (Fermentas) according to the manufacturer’s instructions. Real-time PCR was performed using a StepOnePlus and SYBR Green PCR Master Mix (Applied Biosystems) containing ROX as internal control. For all PCRs the following cycling conditions were used: 10 min at 95°C, 40 cycles of 15 s at 95°C, and 60 s at 60°C. Primers used are given in Table S1 in Supplementary Material. Calculation of expression levels in relation to *AtSAND* (At2g28390) or *AtACT2* (At3g18780) expression was performed using the 2^−ΔΔCt^ method according to Livak and Schmittgen (2001).

### Microarray hybridization and data analysis

Microarray analysis was performed using the ATH1 *Arabidopsis* GeneChip. The quality control of RNA, preparation of biotinylated RNA, and hybridization was performed at KFB Regensburg^1^ using the GeneChip® 3′ IVT Express Kit and Affymetrix standard protocols. All microarray data reported here are described following MIAME guidelines and deposited in NCBI Gene Expression Omnibus (GEO) under the accession number GSE27704.

#### Normalization and differential expression

In order to analyze differential expression among the *Arabidopsis* wild-type Col-0 control, the wild-type seedlings treated with GAB, and the *gun4-1* mutant, microarray signal intensities were calculated from raw data by using robust multi-array average (RMA) expression measure (Irizarry et al., 2003). Subsequently, pairwise comparisons between all wild-type replicates and all GAB-treated replicates or all *gun4-1* replicates were performed to tag differentially expressed genes in treated or mutant seedlings. For this purpose, the LIMMA package (Smyth, 2004) in R/Bioconductor (Gentleman et al., 2004) was used with an empirical Bayes linear modeling approach. The obtained *P*-values for multiple testing were corrected according to Benjamini and Yekutieli (2001). Genes were considered to be significantly differentially expressed if their respective *P*-value was ≤0.05 and the log-fold change was either <log(2/3) or >log(3/2).

#### Heatmaps

A set of these differentially expressed genes was clustered according to their log-fold change using a *k*-means clustering approach in *R* (Hartigan and Wong, 1978; Ihaka and Gentleman, 1996). The expression of each gene in each cluster was depicted using a heatmap implemented in the *R* stats package. The scaling was based on rows, thus left at default, while the colors were set to an interpolation of blue and yellow. The genes being clustered in a Venn diagram were depicted alike.

#### Gene ontology-enrichment analysis

Gene ontology (GO)-enrichment analysis was performed using different algorithms: (i) a customized program was used for calculating over-represented GO terms (Ashburner et al., 2000) in a given set of genes. GO files (released 2010/05) were downloaded from the GO website^2^, and GO annotations for *Arabidopsis* from the TAIR website^3^ (release TAIR9). The subsequent calculation was based on hypergeometric distribution. The resulting *P*-value was corrected using the Bonferroni correction. GO terms with a probability of ≤0.05 were accepted as significant, whereas greater values lead to a rejection of a specific GO term. (ii) The program BiNGO 2.44 with Cytoscape 2.7 (Maere et al., 2005) was used together with the Amigo website^4^. A correction for false discovery rates was determined with BiNGO software using the method of Benjamini and Hochberg (1995).

#### Motif discovery

For motif discovery we used FIRE, Finding Informative Regulatory Elements (Elemento et al., 2007). Based on the concept of mutual information, FIRE calculates putative *cis* elements in a set of genes using a database of promoter elements. Further, FIRE scores simple motif definitions in the form of *k*-mers, searching each combination for under- or over-representation in a defined set of genes. It is possible to define a set of genes using a continuous FIRE approach or using a pre-clustered set as discrete approach. For our analysis, we used a 2-kb upstream promoter annotation based on TAIR9. We further used FIRE in a continuous analysis with a standard robustness index threshold of 6, and with 3–10 *k*-mers.

### Statistical note

All experiments were performed independently three to six times. In order to test the significant differences between calculated values, equality of variances was tested by a *F*-test followed by Student’s *t*-test using a *P*-value of <0.05 as the threshold for significant difference.

## Results

### *Arabidopsis gun* mutants share reduced ALA synthesis rates

**Figure 2:**
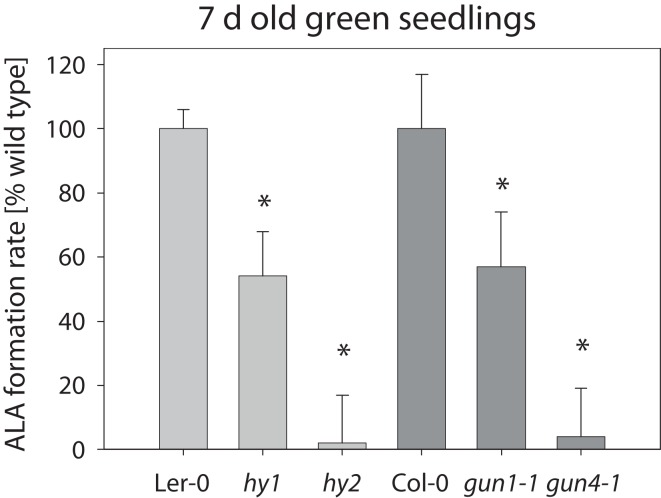
**ALA formation rate in four *Arabidopsis hy1*, *hy2*, *gun1-1*, and *gun4-1* mutants**. The ALA formation rate was determined in the leaves of 7-day-old seedlings grown on 0.5× MS medium supplemented with 1% (w/v) sucrose and given in% of the corresponding wild-type Ler-0 (light gray, 11 pmol ALA mg fw^−1^ h^−1^) or Col-0 (dark gray, 20 pmol ALA mg fw^−1^ h^−1^), respectively. Data are means of at least three biological replica ± SD. * Indicates a significant difference from wild-type at a level of *P* < 0.05.

Inactivation of the expression of Mg chelatase, MgProto methyltransferase, and Chl synthase, enzymes in the Chl-synthesizing branch, results in diminished ALA-forming capacity (Papenbrock et al., 2000b; Alawady and Grimm, 2005; Shalygo et al., 2009). Since all but one known *Arabidopsis* *gun* mutants are also affected in genes related to tetrapyrrole biosynthesis (Nott et al., 2006), we collectively tested the *Arabidopsis* *gun1-1*, *hy1* (allelic to *gun2*), *hy2* (allelic to *gun3*), and *gun4-1* mutants for their *in vivo* ALA synthesis rate. Analysis of 7-day-old *hy1*, *hy2*, and *gun4-1* mutant seedlings revealed a severe reduction in ALA synthesis rate compared to their corresponding wild-types Ler-0 and Col-0 (Figure 2); ALA formation was also reduced in *gun1-1* seedlings. As a consequence of decreased ALA formation, the contents of Chl (a + b) and carotenoids were significantly reduced in 7-day-old *hy1*, *hy2*, *gun1-1*, and *gun4-1* seedlings compared with wild-type seedlings (Table 1).

**Table 1:** **Pigment content of 7-day-old *Arabidopsishy1*, *hy2*, *gun1-1*, and *gun4-1* mutants and the corresponding wild-type seedlings Ler-0 and Col-0**.

Line	Chl (a + b) (ng mg fw^−1^)	Carotenoids (ng mg fw^−1^)
Ler-0	172 ± 29	42 ± 5
*hy1*	102 ± 9*	32 ± 3
*hy2*	68 ± 15*	22 ± 6*
Col-0	207 ± 19	54 ± 5
*gun1-1*	114 ± 19*	36 ± 7*
*gun4-1*	64 ± 7*	22 ± 2*

Given are means ± SD of three independent extractions. *,Indicates a significant difference from the wild-type (*P* < 0.05).

### The *gun* phenotype coincides with the modulation of ALA synthesis rates

Application of 10 μM GAB was reported to diminish Chl accumulation by approximately 50% in de-etiolating barley leaves (Hill et al., 1985; Nair et al., 1990) based on the inhibition of GSAT activity (Grimm et al., 1991), though not affecting the GluTR and GSAT abundance (Demko et al., 2010). In *Arabidopsis* application of 10 μM GAB resulted in an 80% reduction of Chl a and b contents in treated seedlings compared to untreated, de-etiolating seedlings illuminated for 6 h as well as 6-day-old green seedlings (Figure S1A in Supplementary Material). The phenotype of the GAB-treated plants with low pigment contents was similar to the visible phenotype of the *Arabidopsis gun4-1* mutant (Figure S1B in Supplementary Material). Since *gun* mutants and GAB-treated seedlings share the reduced ALA synthesis rate, we were interested to prove whether *Arabidopsis* wild-type seedlings with GAB-inhibited ALA synthesis modulate expression of marker genes for plastid signaling similarly to *gun* mutants.

In parallel to GAB inhibition of ALA biosynthesis, we intended to modify reversely the endogenous pool of ALA by additional supply of ALA to seedlings. It was previously reported that feeding of etiolated seedlings with increasing amounts of ALA correlates with synchronously increasing levels of non-photoconvertible Pchlide (Armstrong et al., 1995). In order to raise the intracellular ALA pool without photo-oxidative risks we performed preliminary experiments to select the appropriate amounts of ALA. Application of ALA to etiolated *Arabidopsis* seedlings caused increasing Pchlide levels when young seedlings were fed with ALA at amounts greater than 50 μM. Application of 100 μM ALA resulted in four-time higher Pchlide levels in etiolated seedlings in the analyzed time period compared to untreated seedlings (Figure S2 in Supplementary Material; 1.0 ± 0.3 vs. 4.5 ± 1.3 pmol mg fw^−1^). Light-grown seedlings fed with 100 μM ALA did not show visible photo-bleaching at the given light exposure (Figure S3 in Supplementary Material), though the addition of ALA at concentrations greater than 250 μM generated a visible stress phenotype in illuminated *Arabidopsis* seedlings (at 100 μmol photons m^−2^ s^−1^) that was likely due to elevated levels of photo-reactive tetrapyrrole intermediates.

**Figure 3:**
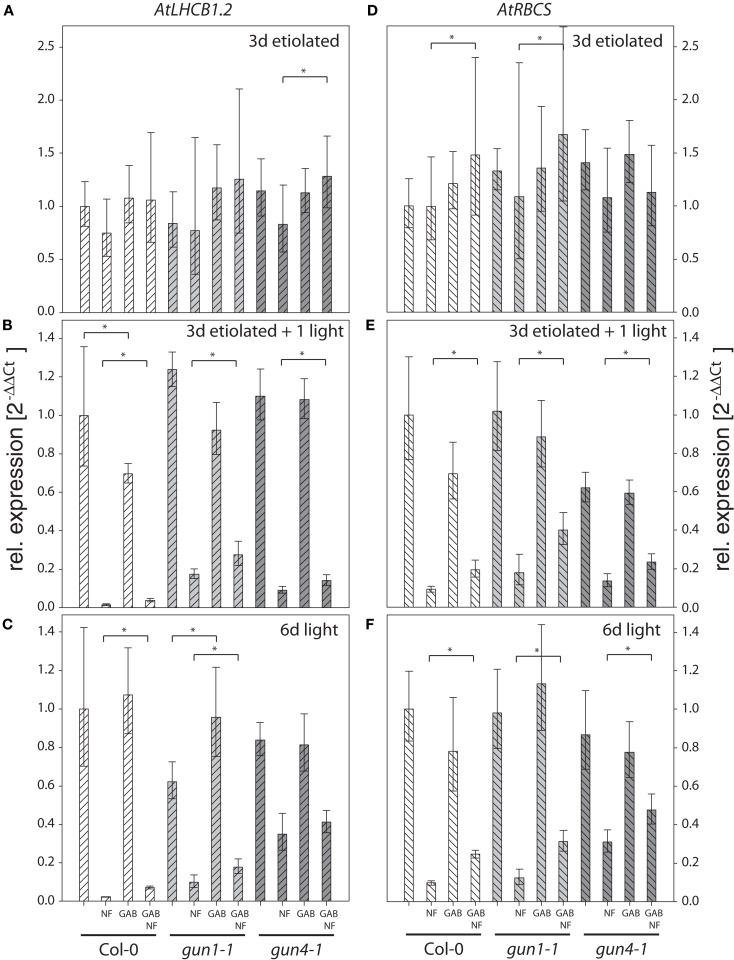
**Quantitative analysis of *AtLHCB1.2* (A–C) and *AtRBCS* (D–F) transcripts in *Arabidopsis* seedlings fed with gabaculine (GAB) and norflurazon (NF)**. Seedlings of *Arabidopsis* wild-type Col-0, *gun1-1*, and *gun4-1* mutants fed with or without 10 μM GAB and 1 μM NF were etiolated for 3 days **(A,D)**, etiolated for 3 days and subsequently illuminated for 1 day **(B,E)**, or germinated in photoperiodic light for 6 day **(C,F)**. *AtLHCB1.2* and *AtRBCS* expression levels were quantified by real-time PCR and calculated by the 2^−ΔΔCt^ method using *AtACT2* expression as standard. Expression data are compared to the untreated wild-type Col-0 and shown as means of at least three biological replicates ± SD. * Indicates a significant difference of pairs indicated by brackets at a level of *P* < 0.05. Tested were GAB-treated samples vs. untreated and GAB and NF treated vs. NF-treated samples within each genotype.

**Figure 4:**
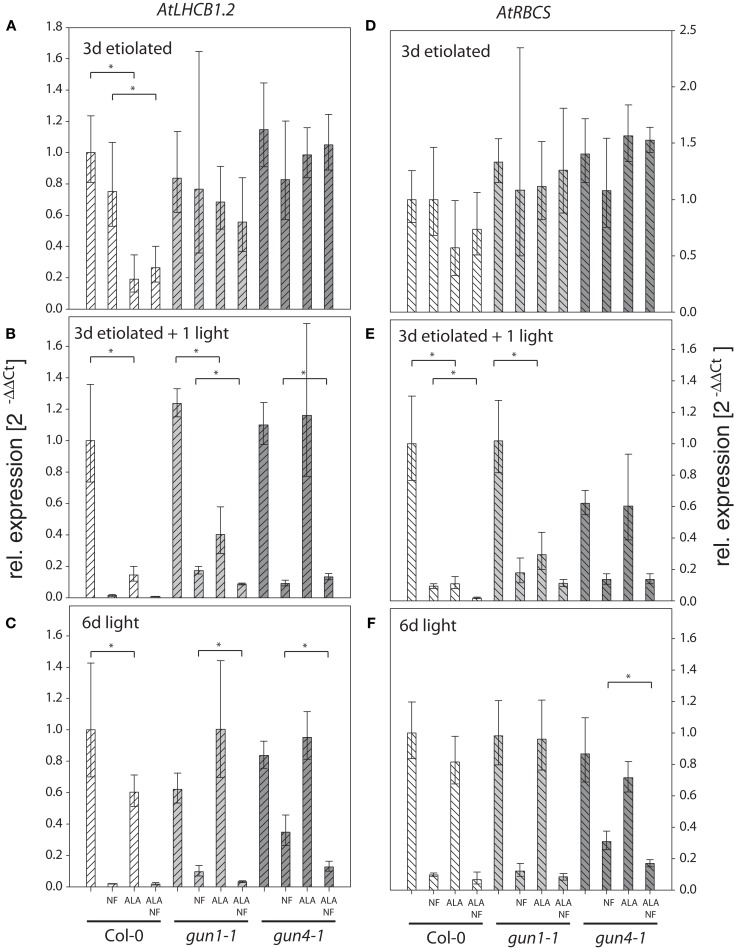
**Quantitative analysis of *AtLHCB1.2* (A–C) and *AtRBCS* (D–F) transcripts in *Arabidopsis* seedlings fed with 5-aminolevulinic acid (ALA) and norflurazon (NF)**. Seedlings of *Arabidopsis* wild-type Col-0, *gun1-1*, and *gun4-1* mutants fed with or without 100 μM ALA and 1 μM NF were etiolated for 3 days **(A,D)**, etiolated for 3 days and subsequently illuminated for 1 day **(B,E)**, or germinated in photoperiodic light for 6 days **(C,F)**. *AtLHCB1.2* and *AtRBCS* expression levels were quantified by real-time PCR and calculated by the 2^−ΔΔCt^ method using *AtACT2* expression as standard. Expression data are compared to the untreated wild-type Col-0 and shown as means of at least three biological replicates ± SD. * Indicates a significant difference of pairs indicated by brackets at a level of *P* < 0.05. Tested were ALA treated samples vs. untreated and ALA and NF treated vs. NF-treated samples within each genotype.

Then, wild-type Col-0, *gun1-1* and *gun4-1* seedlings were subjected to 10 μM GAB or 100 μM ALA during germination and were incubated with NF to replicate experimental conditions that were applied to study the *gun* phenotype. Control and chemically treated wild-type and *gun* mutants were harvested either after 3-day-growth in darkness, 3 days in darkness followed by 1 day light exposure, or after 6 days growth in photoperiodic light (12 h, 100–120 μmol photons m^−2^ s^−1^). The transcription levels of two photosynthesis genes, *AtLHCB1.2* and *AtRBSC*, were determined by qRT-PCR in these samples (Figures 3 and 4).

The analysis of the transcription levels of *AtLHCB1.2* and *AtRBSC* in wild-type Col-0, *gun1-1* and *gun4-1* seedlings subjected to 10 μM GAB during germination and incubated with NF revealed the following results: (i) Inhibition of carotenoid biosynthesis by NF treatment drastically lowered the transcript level in light-exposed, but not in etiolated seedlings (Figures 3 and 4). (ii) The *AtLHCB1.2* and *AtRBCS* expression of NF-treated and light-exposed *gun1-1* and *gun4-1* seedlings was de-repressed relative to that of wild-type Col-0 and confirmed the expected *gun* phenotype in our experimental approaches (Figures 3 and 4). (iii) Inhibition of ALA synthesis by GAB did not affect the expression of *AtLHCB1.2* and *AtRBCS* in etiolated, greening, or green seedlings, with the exception of slightly reduced expression levels of both genes in the wild-type Col-0 and *gun1-1* after 1 day of de-etiolation (Figures 3B,E). Six-day-old, green *gun1-1* seedlings showed elevated *AtLHCB1.2* expression in response to GAB (Figure 3C). (iv) GAB supply of NF-incubated *Arabidopsis* seedlings resulted in elevated *AtRBSC* and *AtLHCB1.2* expression levels compared to seedlings treated solely with NF. This counteracting effect of NF plus GAB vs. NF alone on elevated transcript contents was observed in the wild-type Col-0, but also in *gun1-1* and *gun4-1* in both 1 day illuminated (Figures 3B,E) and 6-day-old green seedlings (Figures 3C,F).

The analysis of *AtLHCB1.2* and *AtRBSC* transcription levels in wild-type Col-0, *gun1-1* and *gun4-1* seedlings subjected to 100 μM ALA during germination and incubated with NF revealed the following results: (v) ALA treatment reduced the *AtLHCB1.2* and *AtRBCS* transcript amounts of wild-type Col-0 seedlings in both light and darkness (Figures 4A–C,E). *Gun1-1* showed a compromised accumulation of transcripts of both genes in de-etiolating seedlings (Figures 4B,E), while *AtLHCB1.2* and *AtRBCS* transcript levels remained stable in ALA-fed *gun4-1* seedlings grown under all experimental conditions (Figure 4). (vi) Combined application of NF and ALA in light-exposed seedlings affected the two analyzed transcript contents even more negatively than the exclusive NF supply (Figures 4B,C,F).

Inhibition of ALA synthesis by GAB in NF-treated wild-type Col-0 led to the increased accumulation of both *AtLHCB1.2* and *AtRBCS* at rates that resembled the *gun* phenotype. The effects of GAB treatment were even more enhanced in the NF-treated *gun1-1* mutant, indicating additive acting mechanisms on NGE in *gun1-1*. However, in comparison with the wild-type Col-0, the modulation of *AtLHCB1.2* and *AtRBCS* transcript accumulation was less pronounced when GAB was added to the NF-treated *gun4-1* mutant (Figure 3).

In contrast to the results observed when GAB was added to inhibit ALA synthesis, ALA addition caused decreasing *AtLHCB1.2* and *AtRBSC* transcript levels in *Arabidopsis* wild-type Col-0 seedlings (Figures 4B,E). Since reduced *AtLHCB1.2* and *AtRBSC* transcript contents were also determined in etiolated seedlings, it can be excluded that photo-oxidative inhibition mainly caused the changes of NGE of photosynthetic genes (see also phenotypes displayed in Figure S3 in Supplementary Material). It is concluded that supply of low and non-phototoxic acting amounts of ALA in NF-treated and light-exposed seedlings abrogates the *gun* phenotype. Thus, regulatory consequences of the ALA synthesis rate and the endogenous ALA pool on NGE can be studied in both experimental approaches without the confounding side effects.

### Global response of NGE to inhibition of ALA synthesis

After etiolated *Arabidopsis* Col-0 seedlings were illuminated, Chl contents increased from zero to 31 ± 3 ng Chl a and 12 ± 4 ng Chl b mg fw^−1^ within 6 h of illumination (Figure S4 in Supplementary Material). After 48 h, the contents of Chl a and b were elevated to 157 ± 17 and 44 ± 6 ng mg fw^−1^, respectively. Carotenoid contents increased from 11 ± 1 in etiolated seedlings to 64 ± 6 ng mg fw^−1^ during 48 h of de-etiolation (Figure S4 in Supplementary Material). Since ALA synthesis is highly regulated and determines the amount of metabolites that are introduced into the branched metabolic pathway of tetrapyrrole biosynthesis, it is likely that the capacity of ALA synthesis has a strong impact on chloroplast biogenesis.

**Figure 5:**
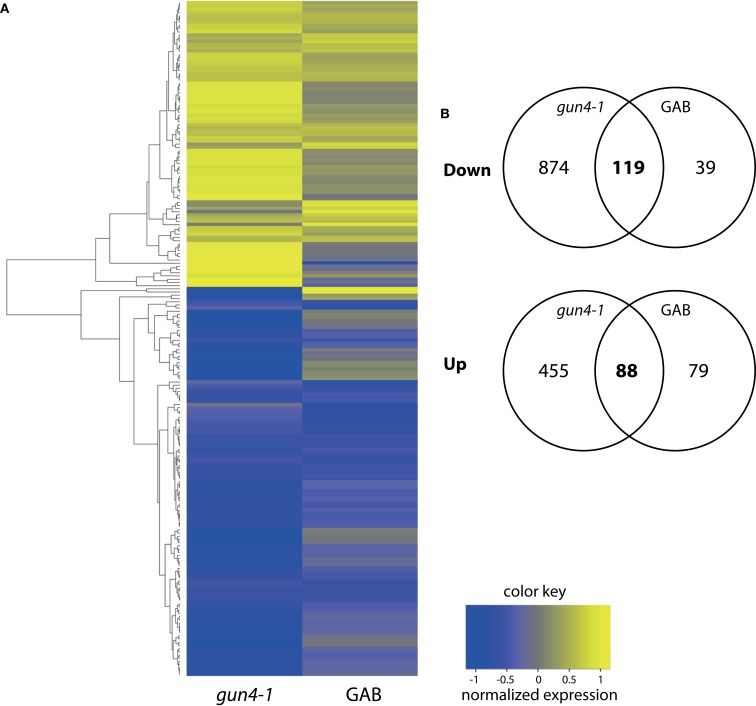
**(A)** Heatmap analysis of differentially expressed genes in GAB-treated Arabidopsis wild-type Col-0 or *gun4-1* mutants 6 h after illumination of etiolated seedlings. All genes showing a fold change of <2/3 or >3/2 and a *P*-value of <0.05 in both conditions were analyzed. **(B)** Venn diagrams summarizing changes of transcript abundance in GAB-treated *Arabidopsis* wild-type Col-0 or *gun4-1* mutants 6 h after illumination of etiolated seedlings. The intersections of the circles represent the number of genes whose transcript abundance was decreased (“Down”) or increased (“Up”) in both compared to the untreated *Arabidopsis* wild-type Col-0.

In continuation of our studies on the regulatory effect of ALA synthesis on NGE, we compared the transcriptome of developing *Arabidopsis* seedlings in response to inhibited ALA synthesis. An Affymetrix GeneChip (ATH1) microarray analysis was performed with RNA from 6 h light-exposed etiolated *Arabidopsis* seedlings incubated with or without 10 μM GAB. Moreover, in comparison to wild-type control we analyzed NGE of the 6 h light-exposed de-etiolating *gun4-1* mutants. The time point for the comparative transcriptome analysis was chosen since initial amounts of Chl accumulated upon illumination of etiolated seedlings (Figure S4 in Supplemental Materials). We decided on *gun4-1*, because the mutant shows posttranslational reduction of ALA synthesis without reduced expression of the ALA-forming enzymes (Peter and Grimm, 2009). We searched for early responsive genes upon inhibited ALA biosynthesis to minimize interference of pleiotropic effects of ALA deficiency (e.g., reduced photosynthetic capacity). Differentially expressed genes were identified in the transcriptome analysis with a *P*-value of ≤0.05 and a log-fold change of <log(2/3) or >log(3/2), respectively, compared to untreated *Arabidopsis* wild-type Col-0 seedlings (Figure 5; Data Sheet 2 in Supplementary Material). Comparison of the transcript profiles revealed that GAB treatment of developing *Arabidopsis* wild-type Col-0 seedlings resulted in the differential regulation of 325 genes representing approximately 1.5% of the entire *Arabidopsis* genome. Inhibited ALA synthesis resulted in the repression of 158 genes and the up-regulation of 167 genes. Compared to wild-type Col-0, *gun4-1* displayed 1536 mis-regulated genes (7.2% of the total ATH1 genome) at the same selected time point of 6 h of de-etiolation, whereas 993 and 543 genes showed a down- and up-regulated expression, respectively (Figure 5; Data Sheet 2 in Supplementary Material). However, a high number of genes were collectively down-regulated (119 genes) and up-regulated (88 genes) in GAB-treated and *gun4-1* seedlings (Figure 5B; Data Sheet 2 in Supplementary Material). The overlapping genes represented 12.5% of the differentially expressed genes (equals 207 combined genes out of the total amount of 1654 differentially regulated genes observed in both microarrays). Apart from two exceptions, all genes were congruently found to be either down- or up-regulated in both sets of seedlings with lower ALA synthesis (GAB-treated and the *gun4-1* seedlings) when compared with control seedlings (Data Sheet 2 in Suppmentary Material). Tables 2 and 3 present the transcripts of the common set of genes in GAB-treated and *gun4-1* seedlings in comparison to untreated wild-type seedlings with the greatest response to inhibition of ALA synthesis based on fold repression (≤0.5, Table 2) and fold induction (≥2, Table 3).

**Table 2:** **Listed are genes that showed a significantly (*P* ≤ 0.05) decreased transcript abundance after 6 h of de-etiolation in response to inhibited ALA formation (GAB) and in the *gun4-1* mutant**.

AGI number	GAB vs. Col-0 fold change	GAB vs. Col-0 *P*-value	*gun4-1* vs. Col-0 fold change	*gun4-1* vs. Col-0 *P*-value	SUBA localization	Description	qRT-PCR performed (see Table S2 in Supplementary Material)
At5g08030	0.247	0.000	0.249	0.000	Mito, Cp, ER, Nuc, Ex, Vac	Glycerophosphoryl diester phosphodiesterase	
At1g13650	0.273	0.000	0.381	0.000	Mito, Perox	Unknown protein	
At2g20570	0.300	0.000	0.351	0.000	Cyt	GPRI1 (GBF’S Pro-rich region-interacting factor 1)	Yes
At5g46690	0.345	0.000	0.344	0.000	Nuc	Beta HLH protein 71 (bHLH071)	
At1g61800	0.347	0.000	0.486	0.000	Cp, Mito, Ex, PM	Glucose-6-phosphate transmembrane transporter (GPT2)	Yes
At3g26960	0.372	0.000	0.288	0.000	Ex, ER, Nuc	Unknown protein	Yes
At5g55450	0.376	0.005	0.278	0.000	Ex, ER	Protease inhibitor	
At4g04610	0.385	0.019	0.166	0.000	Cp	APS reductase 1 (APR1)	Yes
At4g28250	0.387	0.003	0.182	0.000	Ex, ER, Vac	ATEXPB3 (Expansin B3)	Yes
At2g20750	0.407	0.000	0.176	0.000	Ex	ATEXPB1 (Expansin B1)	
At3g61820	0.414	0.000	0.344	0.000	Cp, Ex	Aspartyl protease family protein	Yes
At3g47380	0.431	0.005	0.465	0.002	Ex, Mito, ER, Nuc	Invertase/pectin methylesterase inhibitor	
At1g56650	0.439	0.000	0.355	0.000	Nuc	PAP1 (production of anthocyanin pigment 1); DNA binding/transcription factor	Yes
At5g45650	0.443	0.001	0.356	0.000	Mito, ER, Ex	Subtilase family protein	Yes
At1g24020	0.457	0.017	0.160	0.000	Cyt, Perox	MLP423 (MLP-like protein 423)	Yes
At3g59670	0.459	0.000	0.528	0.000	Nuc	Unknown protein	
At2g31270	0.460	0.000	0.645	0.005	Mito, Nuc, Cp	CDT1A (homolog of yeast CDT1 A); cyclin-dependent protein kinase	
At4g11460	0.468	0.014	0.271	0.000	Ex, ER, Nuc, Vac	Protein kinase family protein	
At4g34588	0.474	0.003	0.428	0.000	No data	CPuORF2 (conserved peptide upstream open reading frame 2)	
At4g34590	0.474	0.003	0.428	0.000	Nuc	GBF6 (G-BOX BINDING FACTOR 6)	Yes
At5g05960	0.477	0.014	0.170	0.000	ER, Ex	Protease inhibitor/seed storage/lipid transfer protein (LTP) family protein	
At4g28680	0.480	0.001	0.432	0.000	Cyt, Mit, ER	Putative tyrosine decarboxylase	
At3g05600	0.481	0.005	0.459	0.001	Cp, Perox, Cyt	Putative epoxide hydrolase	Yes
At4g39510	0.485	0.001	0.369	0.000	Mito, ER, Cyt, Ex, Nuc	CYP96A12; electron carrier/heme binding/iron ion binding/monooxygenase/oxygen binding	Yes
At1g66940	0.493	0.002	0.265	0.000	Ex, ER, Cyt, Ex	Protein kinase-related	Yes
At4g38950	0.500	0.004	0.393	0.000	Nuc	Kinesin motor family protein	Yes

*In contrast to the results shown in Figure 5, there are only transcripts listed with a fold change ≤0.5. Given are the fold changes, corresponding *P*-values, the putative subcellular localizations using the SUBA database (Heazlewood et al., 2007) and gene annotations based on the TAIR9 release of the *Arabidopsis* genome. (Cp, chloroplasts; Cyt, cytosol; ER, endoplasmic reticulum; Ex, extracellular; Mito, mitochondria; Nuc, nucleus; Perox, peroxisome; PM, plasma membrane; Vac, vacuole)*.

**Table 3:** **Listed are genes that showed a significantly (*P* ≤ 0.05) increased transcript abundance after 6 h of de-etiolation in response to inhibited ALA formation (GAB) and in the *gun4-1* mutant**.

AGI number	GAB vs. Col-0 fold change	GAB vs. Col-0 *P*-value	*gun4-1* vs. Col-0 fold change	*gun4-1* vs. Col-0 *P*-value	SUBA localization	Description	qRT-PCR performed (see Table S2 in Supplementary Material)
At1g05560	4.010	0.000	2.057	0.000	Cp, Mito, Cyt	UDP-glucose:4-aminobenzoate acylglucosyltransferase (UGT75B1)	Yes
At1g22890	3.708	0.000	6.331	0.000	ER, Nuc, Cp	Unknown protein	Yes
At2g04040	3.267	0.001	3.156	0.000	PM, Ex, Vac	TX1; antiporter/multidrug efflux pump/multidrug transporter/transporter	
At3g22060	2.838	0.000	3.278	0.000	Ex, Vac	Receptor protein kinase-related	Yes
At2g19800	2.838	0.000	2.686	0.000	Cyt	MIOX2 (Myo-inositol oxygenase 2)	
At5g16980	2.773	0.000	1.635	0.011	Cyt, Mito, Cp	Putative NADP-dependent oxidoreductase	
At3g20340	2.728	0.000	1.903	0.001	Nuc	Down-regulated under photo-oxidative stress.	
At5g43450	2.722	0.000	3.317	0.000	Cyt, Nuc	Putative 2-oxoglutarate-dependent dioxygenase	
At3g47340	2.680	0.000	5.921	0.000	Cyt, Cp	ASN1 (glutamine-dependent asparagine synthase 1)	Yes
At1g05680	2.591	0.005	2.247	0.003	Mito, Cyt, Cp	UDP-glucoronosyl/UDP-glucosyl transferase	Yes
At5g16970	2.590	0.000	1.791	0.000		AT-AER (2-alkenal reductase)	
At3g28740	2.455	0.001	2.472	0.000	Mito, ER, Ex, PM	CYP81D1; electron carrier/heme binding/iron ion binding/monooxygenase/oxygen binding	Yes
At3g20270	2.445	0.011	2.036	0.012	Cp, Cyt, Ex	Lipid-binding serum glycoprotein family protein	
At2g17880	2.423	0.001	3.176	0.000	Cp, Mito, Nuc	Putative DNAJ heat shock protein	Yes
At1g35140	2.362	0.000	12.734	0.000	Ex	Phosphate induced 1 (PHI-1)	
At4g31870	2.340	0.002	2.257	0.001	Cp, Mito, Perox, Nuc	ATGPX7 (glutathione peroxidase 7)	Yes
At1g08630	2.307	0.001	8.617	0.000	Cp, Nuc, Cyt	THA1 (threonine aldolase 1)	Yes
At5g57560	2.218	0.000	6.492	0.000	Ex, Mito, Er	TCH4 (touch 4); hydrolase, acting on glycosyl bonds/xyloglucan:xyloglucosyl transferase	Yes
At5g06860	2.187	0.001	1.775	0.001	Ex	PGIP1 (polygalacturonase inhibiting protein 1)	
At2g29340	2.143	0.001	2.036	0.000	Cyt, Ex, ER	Short-chain dehydrogenase/reductase (SDR) family protein	Yes
At5g17300	2.069	0.001	2.115	0.000	Nuc	Myb family transcription factor	Yes
At4g15260	2.044	0.000	2.059	0.000	Cyt, Cp	UDP-glucoronosyl/UDP-glucosyl transferase	Yes
At5g20230	2.042	0.005	22.472	0.000	PM	ATBCB (blue-copper binding protein)	Yes
At5g14470	2.010	0.001	6.425	0.000	Cyt, Nuc	GHMP kinase-related	
At3g15760	2.005	0.001	2.027	0.000	Ex, Mito, Nuc, Cp	Unknown protein	Yes

*In contrast to the results shown in Figure 5, there are only transcripts listed with a fold change ≥2.0. Given are the fold changes, corresponding *P*-values, the putative subcellular localizations using the SUBA database (Heazlewood et al., 2007) and gene annotations based on the 9.0 release of the *Arabidopsis* genome. (Cp, chloroplasts; Cyt, cytosol; ER, endoplasmic reticulum; Ex, extracellular; Mito, mitochondria; Nuc, nucleus; Perox, peroxisome; PM, plasma membrane; Vac, vacuole)*.

Transcriptomic comparisons showed that a high number of genes commonly affected by reduced ALA synthesis in GAB-treated *Arabidopsis* seedlings were also found in *gun4-1* mutants. Nearly 75% of the genes down-regulated in GAB-treated *Arabidopsis* seedlings were also down-regulated in *gun4-1*. These genes represent 12% of the 993 genes down-regulated in *gun4-1* (Figure 5B). Similarly, 53% of the genes up-regulated in GAB-treated seedlings were also up-regulated in *gun4-1*. These 88 genes represented 16% of the genes up-regulated in *gun4-1*. The results indicate a high number of identical genes identified in both transcriptomic comparisons, which are commonly affected by reduced ALA synthesis in GAB-treated *Arabidopsis* seedlings and *gun4-1*. This set of identical early responsive genes in both transcriptome analyses supports the view that besides the impaired Mg chelatase activity, *gun4-1* is also significantly compromised in ALA biosynthesis. Due to additional effects of GUN4 deficiency on other physiological processes, the transcriptome of *gun4-1* displayed a broader range of modulated genes.

**Figure 6:**
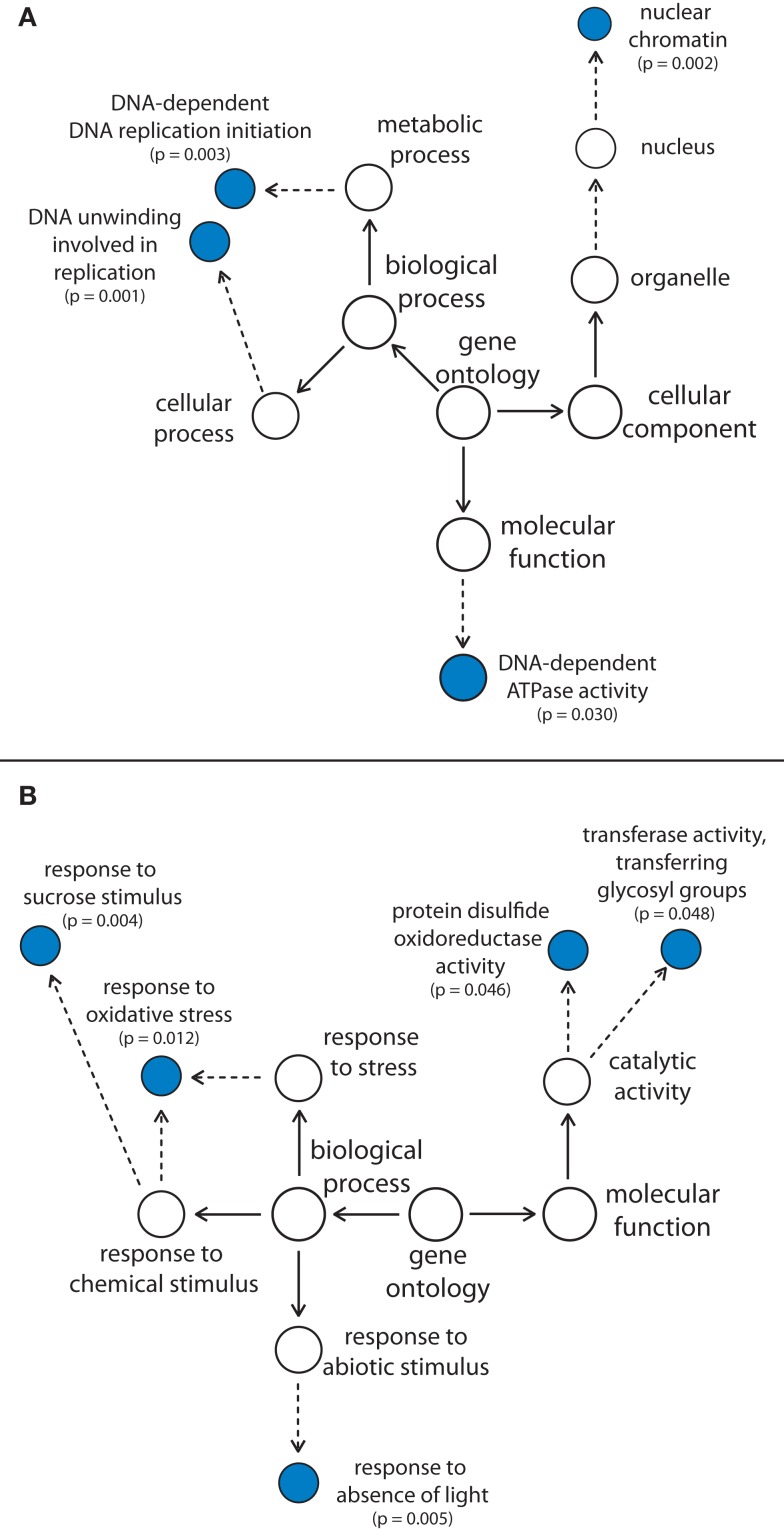
**Overrepresentation of gene ontology (GO) categories in genes showing decreased (A) and increased (B) transcript abundance in GAB-treated *Arabidopsis* wild-type Col-0 and *gun4-1* mutants 6 h after illumination of etiolated seedlings**. The 119 or 88 genes, respectively, being differentially regulated in both GAB-treated and *gun4-1* seedlings, were analyzed for over-represented GO categories (*P*-value of <0.05). Dotted lines indicate intermediate categories that are not shown.

Analysis of GO categories revealed an overrepresentation of transcripts encoding proteins involved in nuclear DNA replication within the cluster of genes being down-regulated in both GAB-treated *Arabidopsis* wild-type Col-0 and *gun4-1* seedlings (Figure 6A). Within the cluster of up-regulated genes, neither cellular components nor functions were overrepresented. Genes encoding proteins responding to sucrose stimulus, oxidative stress, and the absence of light were overrepresented within the biological process category, whereas transcripts involved in posttranslational modification of proteins (disulfide bonds and glycosyl groups) were overrepresented in the molecular function category (Figure 6B). GO analyses extended to all differentially expressed transcripts additionally revealed that reduction of ALA synthesis in GAB-treated seedlings and in *gun4-1* mutants caused a reduced abundance of transcripts for sulfur metabolism (sulfate assimilation, sulfate reduction, and adenyl-sulfate reductase activity) at an early stage of development (Figure S5A in Supplementary Material). The significantly over-represented GO categories additionally identified in up-regulated transcripts were more diverse and include transcripts related to the circadian rhythm, redox homeostasis, auxin signaling, and tocopherol biosynthesis (Figure S5B in Supplementary Material).

**Figure 7:**
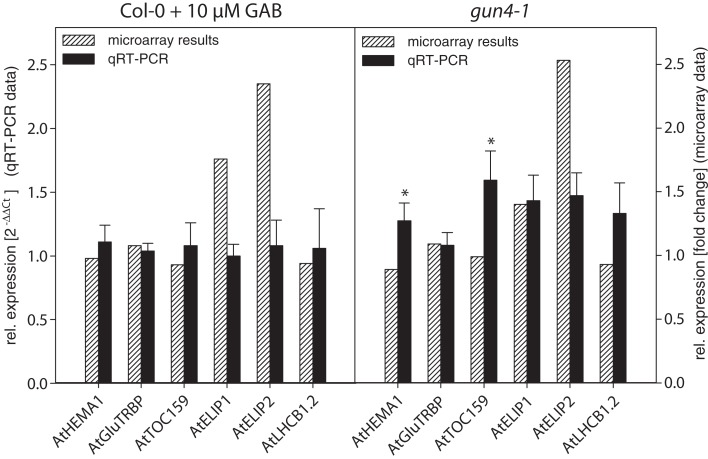
**Comparison of expression data obtained by microarray analysis and qRT-PCR**. RNA from GAB-treated *Arabidopsis* wild-type seedlings and *gun4-1* mutant seedlings was extracted 6 h after light exposure of 3-day-old etiolated seedlings. In addition to microarray analysis, transcript abundance of a defined set of genes was determined by quantitative real-time PCR and calculated in relation to *AtSAND* expression compared to untreated *Arabidopsis* wild-type Col-0 seedlings [2^−ΔΔCt^]. qRT-PCR data are shown as average ± SD of three biological replicates. * Indicates a significant difference from the untreated *Arabidopsis* wild-type Col-0 (*P*-value of <0.05).

It is remarkable that within the small set of 207 combined genes that differentially respond to two different mechanisms of inhibited ALA synthesis (Figure 5B), no genes were found which are involved in Chl biosynthesis and photosynthesis (Tables 2 and 3; Data Sheet 2 in Supplementary Material). We compared microarray data with transcripts levels obtained by qRT-PCR of two genes involved in ALA synthesis, *AtHEMA1* and *AtGluTRBP* (Czarnecki et al., 2011a) and other marker genes for chloroplast functions, e.g., *AtTOC159*, *AtELIP1*, *AtELIP2*, and *AtLHCB1.2* in 6 h de-etiolated GAB-treated wild-type and *gun4-1* seedlings. In both sets of seedlings the expression levels of these genes were not significantly altered in response to inhibition of ALA formation (Figure 7; Table S2 in Supplementary Material), except a 30–50% stimulated expression of *AtHEMA1* and *AtTOC159* in *gun4-1* compared to the control transcript levels of wild-type seedlings.

**Figure 8:**
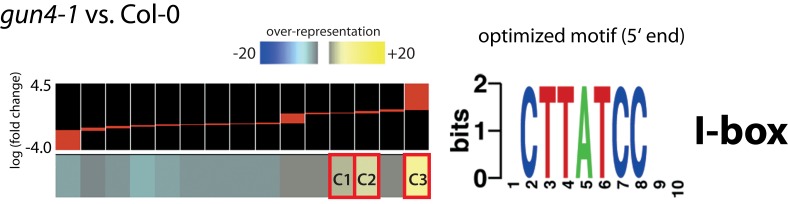
**Identification of informative promoter sequence motifs of regulated transcripts in *gun4-1* mutant seedlings compared to untreated *Arabidopsis* wild-type Col-0 seedlings**. Columns on the left correspond to groups of genes clustered according to the similarity in their expression pattern [log (fold change)] within the microarray experiments. Clusters highlighted in red (C1, C2, and C3) show a significant over-representation of the specified motif. The sequence on the right represents the optimized motif and its name based on JASPAR, TRANSFAC, or PLACE. Names and descriptions of transcripts forming clusters C1–C3 were given in Data Sheet 3 in Supplementary Material.

Screening of genomic sequences upstream of the transcription initiation region of differentially expressed genes in GAB-treated or *gun4-1* *Arabidopsis* seedlings using FIRE analysis did not reveal an over-representation of a promoter motif in GAB-treated *Arabidopsis* seedlings. However, an I-box motif (5′-CTTATCC-3′) was overrepresented in three clusters representing transcripts with enhanced expression in *gun4-1* seedlings (Figure 8; Data Sheet 3 in Supplementary Material). Consistently, over-represented GO categories were identified within genes forming clusters with over-represented promoter motifs. The I-box motif found in clusters C1–C3 formed by genes over-expressed in *gun4-1* seedlings seems to be a promoter element of genes involved in sucrose and light stress, but also secondary metabolic systems, e.g., cell wall biosynthesis and defense reactions (Table 4).

**Table 4:** **Cluster C1–C3 (Figure 8) showing significant over-represented specific motifs in mis-regulated genes of *gun4-1* mutants analyzed for overrepresentation of gene ontology (GO) categories**.

Cluster	Domain (C, F, P)	*P*-value	GO category	Description
C1	F	0.014	GO:0016491	Oxidoreductase activity
C2	P	0.004	GO:0009743	Response to carbohydrate stimulus
		0.032	GO:0010200	Response to chitin
C3	C	0.037	GO:0044464	Cell part
	F	0.047	GO:0005199	Structural constituent of cell wall
		0.036	GO:0016762	Xyloglucan:xyloglucoyl transferase activity
	P	0.023	GO:0050832	Defense response to fungus
		0.006	GO:0009646	Response to absence of light
		0.026	GO:0010200	Response to chitin
		0.005	GO:0009744	Response to sucrose stimulus

*The genes forming the respective clusters (Data Sheet 3 in Supplementary Material) were analyzed for over-represented GO categories (*P* ≤ 0.05). Intermediate GO categories are omitted. C, cellular component; F, molecular function; P, biological process*.

**Figure 9:**
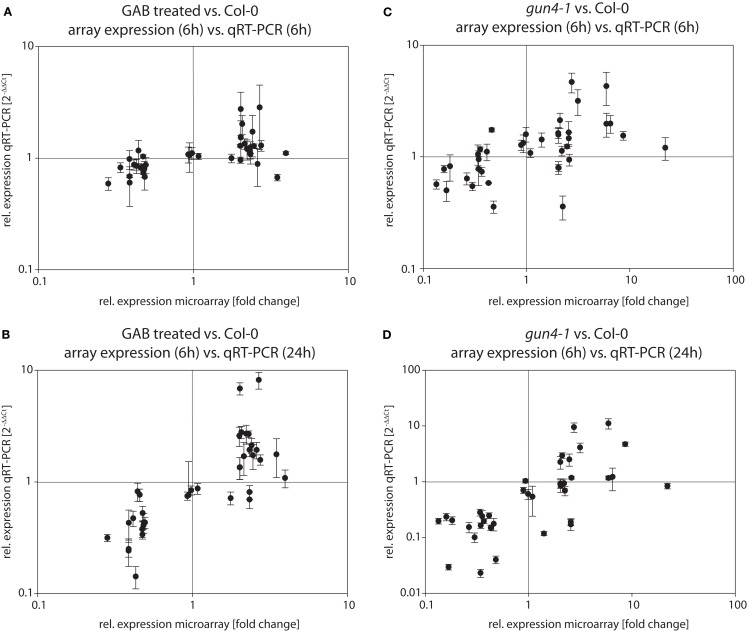
**Verification of microarray results via qRT-PCR**. RNA from GAB-treated *Arabidopsis* wild-type seedlings **(A,B)** and *gun4-1* mutant seedlings **(C,D)** was extracted 6 h **(A,C)** and 24 h **(B,D)**, respectively, after light exposure. Transcript abundance of a defined set of genes (see Tables 2 and 3 and Table S2 in Supplementary Material) was determined by quantitative real-time PCR and calculated as relative expression [2^−ΔΔCt^] compared to untreated *Arabidopsis* wild-type Col-0 seedlings. Relative expressions obtained by qRT-PCR are plotted against relative expressions in microarray experiments (sampling 6 h after light exposure) in order to compare relative expressions in both experimental approaches.

Quality and robustness of expression data obtained by microarray analysis were validated by independent expression analysis of selected genes via qRT-PCR. Therefore, 36 genes up- or down-regulated in both *gun4-1* and GAB-treated wild-type seedlings were selected either by reason of the putative chloroplast localization of their encoded proteins, their function as transcription factor, or the strong alterations in the transcript abundance determined by the microarray analysis in comparison to control (Table S2 in Supplementary Material). Quantitative RT-PCR expression analysis was performed with de-etiolated seedlings after 6 and 24 h of illumination. The data of the global transcriptome analysis were confirmed in approximately 67% of the performed qRT-PCR reactions. Moreover, the majority of transcripts being mis-regulated in GAB-treated wild-type and *gun4-1* seedlings after 6 h of de-etiolation were also mis-regulated after 24 h of light exposure (Figure 9; Table S2 in Supplementary Material). This confirmation revealed a robust data set created by microarray analysis of transcriptional changes in response to reduced ALA synthesis.

## Discussion

### Compromised ALA biosynthesis is a trait of *gun* mutants

By using GAB to reduce ALA formation we proved a role of ALA-synthesizing activity in plastid-to-nucleus communication and modulation of NGE. Transcript accumulation of *LHCB1.2* has been previously examined in NF-treated *Arabidopsis* seedlings to identify mutants affected in retrograde signaling (Susek et al., 1993). Four out of five initially identified *gun* mutants are perturbed in tetrapyrrole biosynthesis. We found that reduced ALA synthesis rates are a common metabolic trait of *gun1-1*, *hy1* (allelic to *gun2*), *hy2* (allelic to *gun3*), and *gun4-1* mutants (Figure 2). A lower ALA-synthesizing capacity has been previously reported for *gun4-1* (Peter and Grimm, 2009), and other transgenic lines with reduced expression of *CHLH*, *CHLI*, *CHLM*, and *CHLG* encoding either one of the three different Mg chelatase subunits, Mgproto methyltransferase, or Chl synthase, respectively (Figure 1; Papenbrock et al., 2000a,b; Alawady and Grimm, 2005; Shalygo et al., 2009). Mutants with deficiency in the synthesis of Mg chelatase subunits are considered to be phenotypically equivalent to the *gun5* mutant.

We added GAB to *Arabidopsis* wild-type seedlings to simulate reductions in ALA synthesis and changes in NGE of the *gun* mutants. We showed that inhibition of ALA formation by GAB mimics the *gun* phenotype in NF-treated *Arabidopsis* wild-type seedlings and results in elevated transcript levels of the photosynthetic genes *LHCB1.2* and *RBCS* compared to the NF treatment without GAB addition (Figure 3). GAB addition had similar effects on NF-treated *gun1-1* and *gun4-1* seedlings (Figure 3). In contrast to NF-treated and light-exposed seedlings, the combined addition of NF and GAB did not influence the transcript levels of *LHCB1.2* and *RBCS* in etiolated *gun*-mutant and wild-type seedlings. The lack of an effect of GAB addition on NGE in etiolated seedlings is likely explained by a minimal ALA synthesis rate caused by FLU-mediated inactivation of GluTR (Meskauskiene et al., 2001; Meskauskiene and Apel, 2002; Richter et al., 2010; Kauss et al., 2012).

### Additional ALA supply and accumulation of photosynthetic genes

Since reduced ALA synthesis was shown to mimic a *gun* phenotype in wild-type seedlings, we then added low amounts of ALA to germinating *Arabidopsis* *gun1-1* and *gun4-1* seedlings to test if elevated ALA amounts suppress the *gun* phenotype. Additional supply of low amounts of ALA decreased the transcript levels of *LHCB1.2* and *RBCS* in both etiolated and illuminated wild-type seedlings. ALA feeding potentiated the NF-induced decrease of transcript contents of photosynthetic genes in greening and green wild-type and *gun1-1* seedlings. In contrast, elevated ALA pools in *gun4-1* resulted in less pronounced changes of NGE compared to both wild-type Col-0 and *gun1-1*, indicating that *gun4-1* is less sensitive to exogenously applied ALA (Figure 4).

It was previously reported that ALA feeding of etiolated *Arabidopsis* seedlings results in an increased *LHCB* promoter-driven GUS-activity (Lopez-Juez et al., 1999), whereas the same amount of exogenous ALA leads to suppressed *LHCB* expression in dark-incubated and light-exposed seedlings (Vinti et al., 2000). An inhibition of the light signaling-mediated induction of gene expression was proposed in both cases (Lopez-Juez et al., 1999; Vinti et al., 2000). We also observed the reduction in *LHCB* and *RBCS* mRNA levels in wild-type and *gun1-1* seedlings as a response to ALA feeding (Figure 4), but cannot confirm a stimulatory effect of ALA on *LHCB1.2* expression in etiolated seedlings. The difference might be explained with different concentrations of ALA used or different developmental states of the seedlings (3 vs. 7 days dark incubation).

As feeding of *Arabidopsis* wild-type, *gun1-1* and *gun4-1* mutants with exogenous ALA represses the *gun* phenotype under NF treatment (Figure 4), we suggest that the observed changes in expression of *LHCB1.2* and *RBCS* are either due to modification of ALA biosynthesis rates or the ALA pool. This is consistent with the observed changes in the accumulation of these mRNAs in response to applied NF and GAB (Figure 3). Considering the opposite effects of ALA supply and inhibition of ALA synthesis, a direct impact of ALA biosynthesis or the ALA steady-state level is proposed.

It is remarkable that transcript accumulation of the *LHCB1.2* and *RBCS* in the NF-treated *gun4-1* mutant showed a lower sensitivity to the addition of GAB and ALA. This favors the idea of GUN4 being a component of the mechanism translating the state of plastid ALA synthesis into a nuclear response. A role of GUN4 in posttranslational regulation of ALA formation has been previously proposed (Peter and Grimm, 2009). It is hypothesized that GUN4 stimulates ALA synthesis in light. This is supported by observations that *gun4* knock-out mutants cannot survive photoperiodic growth conditions and do not accumulate Chl under these conditions, but slowly green and grow under continuous dim light.

However, although we clearly show that GAB treatment mimics the *gun* phenotype in *Arabidopsis* wild-type seedlings and excess ALA represses the *gun* phenotype of NF-treated *gun* mutants, it should be kept in mind that NF generally is a very deleterious herbicide leading to a bleaching leaf phenotype initially caused by inhibition phytoene desaturase, an enzyme involved in carotenoid biosynthesis (Breitenbach et al., 2002). It was previously suggested that retrograde signals emitted by chloroplast destruction under NF treatment are heavily influenced by production of a complex ROS pattern that likely acts as a modulator of NGE itself (Op den Camp et al., 2003; Apel and Hirt, 2004; Kim et al., 2008; Moulin et al., 2008).

Considering recently developed ideas that retrograde signaling is not necessarily the result of single and distinct emitted signal molecules but rather the result of continuously sensed metabolic and genetic activities in the organelles (Pogson et al., 2008; Kleine et al., 2009; Pfannschmidt, 2010), changes in NGE provoked by NF-induced inhibition of chloroplast development may not reflect signaling pathways under non-destructive or non-lethal conditions. In this context, it is an important observation that 6-day-old low light-exposed *gun* mutants do not display significantly increased *LHCB1.2* expression under NF treatment (Voigt et al., 2010). Moreover, the authors also found that under NF treatment *LHCB1.2* expression was not increased in several *Arabidopsis* T-DNA insertion mutants affected in genes of tetrapyrrole biosynthesis. In consequence, the appropriateness of PhANGs (e.g., *LHCB1.2*, *RBCS*, *CA*) may put into question as primary target genes of plastid signaling.

### Inhibition of ALA biosynthesis induces changes in the *Arabidopsis* transcriptome

We intended to examine the hypothesis that reduced ALA formation instantaneously releases a retrograde signal and performed a transcriptome analysis to explore rapid changes in transcript profiles in response to lower ALA biosynthesis before other regulatory mechanisms of successive changes in tetrapyrrole biosynthesis and chloroplast biogenesis proceed (e.g., photosynthesis, redox poise, ROS). The microarray analysis with GAB-treated seedlings and *gun4-1* seedlings revealed a common set of significantly up- and down-regulated genes in response to reduced ALA synthesis (Figure 5; Tables 2 and 3). In addition to previously proposed signaling derived from tetrapyrroles (Mochizuki et al., 2001; Strand et al., 2003; Woodson et al., 2011), this list of deregulated genes indicates the potential of ALA synthesis-derived retrograde signaling.

Kobayashi et al. (2009) opened the view on the interrelation between tetrapyrrole biosynthesis, function of tetrapyrrole intermediates, and the cell cycle process involved in DNA replication. The authors found a strong interdependence of plastid and mitochondrial DNA replication prior to nuclear DNA replication in the primitive red alga *Cyanidioschyzon merolae*. The coordination of nuclear DNA replication followed after the organellar replication was regulated through tetrapyrrole signaling contributing to organelle-nucleus communication. In this context it is remarkable to identify groups of over-represented GO categories among genes deregulated in seedlings with reduced ALA-synthesizing capacity that are involved in DNA replication and nuclear chromatin organization (Figure 6A). Moreover, the regulatory dependency of Mg porphyrins and plastid function of GUN1, a pentatricopeptide-repeat protein with the potential to bind nucleic acids implicates a regulatory link between tetrapyrrole biosynthesis and control of genes for DNA and RNA metabolism (Koussevitzky et al., 2007).

### What commends ALA synthesis to be a signal emitter for NGE?

Plastid signals have been proposed to convert multiple light signaling pathways controlling PhANGs (Ruckle et al., 2007). The over-representation of target genes reacting to abiotic stimuli and absence of light indicate a role of reduced ALA synthesis for modulation of light signaling networks (Figure 6B). It is conceivable that the metabolic pathway of Chl biosynthesis contributes to intracellular communication between chloroplast and nucleus in response to environmental and biological cues, such as abiotic stress and sucrose status to adjust the significant energy-converting processes of photosynthesis. In this context, the *cis*-element I-Box was found to be overrepresented in promoters of deregulated genes in *gun4-1* (Figure 8). On the other hand, among deregulated genes carrying an I-box in the promoter region genes responding to sucrose and light stress are overrepresented (Table 4). This finding is in agreement to previous results of I-Box-binding factors that are regulated by light, sugar sensing, and the circadian clock (Donald and Cashmore, 1990; Borello et al., 1993).

The compilation of up- and down-regulated genes identified in the transcriptomes of GAB-treated seedlings as well as *gun4-1* knock-down seedlings revealed a significant and representative set of identically regulated genes indicating that modified ALA biosynthesis and/or the ALA pool in plastids have an impact on NGE at early stages of plant development. Since the time point chosen in our comparative transcriptome analysis corresponded to the first detectable accumulation of Chl (Figure S4 in Supplementary Material) upon illumination of etiolated seedlings, it may be assumed that the described changes in NGE are primary effects in response to inhibited ALA formation. Moreover, typical marker genes for photosynthesis and chloroplast biogenesis are missing among these genes identified in both experimental approaches (Figure 7).

It can be concluded that our experimental setup allows an insight in the primary impact of modulated ALA biosynthesis on NGE. Consecutively, compromised tetrapyrrole biosynthesis will induce numerous regulatory adaptations to long-term perturbation of ALA biosynthesis, including modification of PhANGs expression and additional protective mechanisms against photo-oxidative damage (Op den Camp et al., 2003). This holds true for mutants with impaired tetrapyrrole biosynthesis, such as several *gun* mutants, which experience a reduced ALA synthesis followed by extenuated Chl contents. When NF-treated seedlings are short of carotenoids, Chl-binding proteins cannot be stabilized, free Chl accumulates and causes severe photo-oxidative damage in wild-type seedlings. It is suggested that reduced metabolic flow in the tetrapyrrole biosynthetic pathway will modify the potential risk of photo-oxidative stress. This is in agreement with biochemical and genetic investigations of NF-treated *gun* mutants which point to an integrative plastid signaling converging at least signals derived from tetrapyrrole biosynthesis, organellar gene expression, and abscisic acid signaling (Voigt et al., 2010).

**Figure 10:**
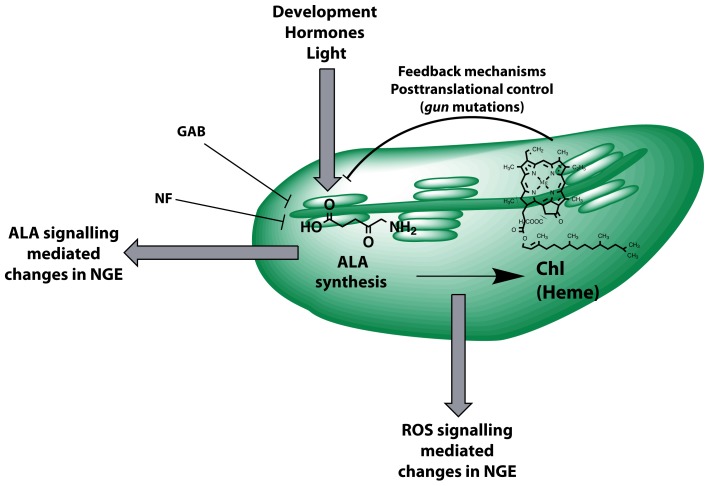
**Model summarizing control mechanisms that act on and are emitted from ALA biosynthesis**. The contribution to plastid-derived retrograde signaling is highlighted. ALA formation is posttranslationally controlled by several known feedback mechanisms of the Chl and Mg branch of the pathway (e.g., heme, Mg chelatase assembly, GUN4) also reflecting the impact of the *gun2*-*gun5* mutations on ALA synthesis. Intermediates of the tetrapyrrole biosynthetic pathway (e.g., Mg Protoporphyrin IX) were discussed to be involved in signaling pathways and likely act via reactive oxygen species (ROS)-mediated changes in nuclear gene expression (NGE). Gabaculine (GAB) directly inhibits ALA formation resulting in changes of NGE at very early stages of chloroplast development. Norflurazon (NF) inhibits tetrapyrrole biosynthesis indirectly.

We propose an extended concept of tetrapyrrole-mediated retrograde signaling that moves an ALA synthesis-dependent mechanism controlling NGE into focus (Figure 10). This model integrates changes in NGE of many mutants affected in tetrapyrrole biosynthesis (Mochizuki et al., 2001; Larkin et al., 2003; Woodson et al., 2011) and takes into account the deleterious properties of intermediates of the Mg branch of tetrapyrrole biosynthesis in plastid-to-nucleus communication (Mochizuki et al., 2008; Moulin et al., 2008; Kleine et al., 2009). This ALA-dependent signaling occurs apart from GUN1-mediated-signaling (Koussevitzky et al., 2007) since regulatory impact of feeding with ALA or GAB on *LHCB1.2* and *RBCS* expression acts additively to the *gun1-1* phenotype (Figures 3 and 4). Since we observed that ALA feeding did not suppress expression of *LHCB1.2* and *RCBS* in *gun4-1*, a function of GUN4 in the retrograde signaling pathway from ALA biosynthesis is suggested (Figure 4). Because multiple endogenous and environmental factors control the rate-limiting ALA synthesis and, thus, control the metabolic flow of tetrapyrrole intermediates for Chl and heme synthesis, retrograde signals emitted at the level of ALA formation are proposed to adjust the transcriptional machinery in the nucleus.

## Conflict of Interest Statement

The authors declare that the research was conducted in the absence of any commercial or financial relationships that could be construed as a potential conflict of interest.

## Supplementary Material

The Supplementary Material for this article can be found online at http://www.frontiersin.org/Plant_Physiology/10.3389/fpls.2012.00236/abstract

Supplementary Table S1 **PCR Primers to amplify gene specific probes used for quantitative real-time PCR**. Primers for *AtLHCB1.2*, and *AtRBCS* were taken from Mochizuki et al. (2008).

Supplementary Table S2 **Verification of microarray results via qRT-PCR**. RNA from GAB-treated *Arabidopsis* wild-type seedlings and *gun4-1* mutant seedlings was extracted 6 and 24 h, respectively, after light exposure. Transcript abundance of a defined set of genes was determined by quantitative real-time PCR and calculated as relative expression (2^−ΔΔCt^) compared to untreated *Arabidopsis* wild-type Col-0 seedlings. qRT-PCR data are given as average ± SD of three biological replicates. Listed are relative expressions obtained by microarray experiments (sampling 6 h after light exposure) as well as qRT-PCR in order to compare relative expressions in both experimental approaches. Relative expressions of qRT-PCR data were calculated using *AtSAND* (for 6 h values) and *AtACT2* (for 24 h values) as reference. Bold: Relative transcript abundance in GAB-treated or *gun4-1* seedlings (determined by qRT-PCR) significantly differs from the untreated *Arabidopsis* wild-type Col-0 (*P* < 0.05).

Supplementary Figure S1 **Pigment content (A) and phenotype (B) of *Arabidopsis* wild-type Col-0 seedlings, Col-0 seedlings treated with 10 μM gabaculine (GAB), and *gun4-1* mutant seedlings 6 h after de-etiolation or after 6 days growing on MS medium (control) or MS medium + 10 μM GAB**. The contents of Chl a, Chl b, and Car of the untreated controls were 0.7 ±  0.2, 0.4 ± 0.2, and 2.6 ± 0.6 ng mg fw^−1^ 6 h after deetiolation and 172 ± 20, 44 ± 3, and 57 ± 5 ng mg fw^−1^ for 6 days old seedlings, respectively.

Supplementary Figure S2 **Pchlide content of etiolated *Arabidopsis* Col-0 seedlings grown in liquid MS medium containing increasing concentrations of ALA**. Surface sterilized seedlings were germinated and grown for 3 days in darkness under continuous shaking. Above an ALA concentration of 50 μM in the medium seedlings over-accumulate Pchlide.

Supplementary Figure S3 **Phenotypes of *Arabidopsis* wild-type Col-0, *gun1-1*, and *gun4-1* treated with combinations of 1 μM norflurazon (NF), 10 μM gabaculine (GAB), and 100 μM ALA, respectively**. Seedlings were grown on 0.5 MS medium supplemented with 1% (w/v) sucrose containing the indicated chemicals for 6 days under photoperiodic growth light (12 h light/12 h dark) at 100–120 μmol photons m^−2^ s^−1^.

Supplementary Figure S4 **Pigment accumulation during de-etiolation of 3-day-old etiolated *Arabidopsis* Col-0 seedlings**. Given are means ± SD of three independent extractions.

Supplementary Figure S5 **Overrepresentation of gene ontology (GO) categories in genes showing decreased (A) and increased (B) transcript abundance in GAB-treated *Arabidopsis* wild-type Col-0 and/or *gun4-1* mutants 6 h after illumination of etiolated seedlings**. All 1032 genes being down-regulated **(A)** and 622 genes being upregulated **(B)** in both GAB treated and *gun4-1* seedlings, were analyzed for overrepresented GO categories (*P*-value of <0.05). Dotted lines indicate intermediate categories that are not shown. Calculation of overrepresented GO categories based on BiNGO 2.44.

## Footnotes

^1^http://www.kfb-regensburg.de

^2^http://www.geneontology.org

^3^http://www.arabidopsis.org

^4^http://amigo.geneontology.org

## Author Note

The submitted manuscript has been authored by a contractor of the U.S. Government under contract DE-AC05-00OR22725. Accordingly, the U.S. Government retains a non-exclusive, royalty-free license to publish or reproduce the published form of this contribution, or allow others to do so, for U.S. Government purposes.

## Abbreviations

ALA, 5-aminolevulinic acid; Chl, chlorophyll; fw, fresh weight; GAB, gabaculine; GluTR, glutamyl tRNA reductase; GO, gene ontology; GSAT, glutamate 1-semialdehyde aminotransferase; MgProto, Mg protoporphyrin IX; MgProtoME, Mg protoporphyrin IX monomethylester; NF, norflurazon; NGE, nuclear gene expression; Pchlide, protochlorophyllide; PhANGs, photosynthesis-associated nuclear genes; POR, protochlorophyllide oxidoreductase; Proto, protoporphyrin IX; ROS, reactive oxygen species.
