# Supplementary material for: Evidence for a Contribution of ALA Synthesis to Plastid-To-Nucleus Signaling
Source: Front Plant Sci. 2012 Oct 29;3:236. doi: 10.3389/fpls.2012.00236 (PMC3483025; doi:10.3389/fpls.2012.00236)
Supplement: Supplementary Table S1 — PCR Primers to amplify gene specific probes used for quantitative real-time PCR. Primers for AtLHCB1.2, and AtRBCS were taken from Mochizuki et al. (2008). [file DataSheet_1.PDF]

Table S1: PCR Primers to amplify gene specific probes used for Northern hybridization and quantitative Real Time PCR**.** Primers for *AtLHCB1.2*, and *AtRBCS* were taken from Mochizuki et al. (2008): The steady-state level of Mg-protoporphyrin IX is not a determinant of plastid-to-nucleus signaling in Arabidopsis. *Proc. Natl. Acad. Sci. U. S. A.* 105**,** 15184-15189.

Gene	Forward and reverse Primer sequence (5' – 3')	Gene	Forward and reverse Primer sequence (5' – 3')
At1g05560	ACTCATAGAAGGGAAACGAC<br>GCTATCTTCTCAATCTCTGTCTC	At3g22840	ATAAGCGTTGAGTCCAAGTCC<br>TCCCTTAGACGAGTGTCCCA
At1g05680	AATGATTTGCCGTCTTTCCT<br>AAACCGTAGTTCTTGTCTTCAG	At3g28740	CTCAACATGGGTTTGTGAAAGTGG<br>GGCAATTAGCTGTCTCACGTG
At1g08630	AAGACTTACGGCTGAGAAACTG<br>CATTGCTTGCTGAAAGCAAGAC	At3g47340	CCAAGATGATCAAGCCAGAG<br>CCAACACCATCACTGAACTG
At1g22890	GCAACCTACGTAACAATTTGC<br>TTGTTTATGGGCCTTGTCCT	At3g48360	AAGAGGAGGAATAACGATAAGGG<br>TTTCCTAAATTGCCTGCAGAG
At1g24020	CTCAGGAACTTCAATGTCTTGTC<br>TAGTCATCCAAGCAGTGGTC	At3g61820	GGCGAGTAATTATCTGATCCC<br>ACCTTGTTGCTGTATGTTTCC
At1g26250	CCTTATGTCTATACTTCACCACC<br>GAGGAGGCTTGTAAACATATGG	At4g04610	CATTGCATTTAGTGGTGCAG<br>GATTCAACCTCCCTGTATCC
At1g56650	GCTGCGAAAAGGTGCTTGGACTAC<br>CCCAGCTCTTACAGGAACTTGGTG	At4g14690	CAAGCCTAAGGTGAGTACGA<br>CGAATCCAACCATTGCTAGTC
At1g61800	ACTTTGTCTGGTGGGTAGTG<br>GAAATCCGCTTCATCGTATTACC	At4g15260	GGAACGGACTTTGGATAGAG<br>CCCACAATGAGTAACAAACCC
At1g66940	CCAGGAGTTAAATGTCGTCC<br>TATTAGATTTGCCCGAACTGC	At4g28250	CTAATCCCAGTCATTTATCGCC<br>GTCTCCTTCTCCATCTTCAAACTC
At2g17880	GGAATTGGGAAACCGATCAG<br>ACGATAAACCGTGTTGATCC	At4g31870	ATACCCTCCCACAACTTCTC<br>GCAAGCAACTTCTGGATGTC
At2g19800	AGACAATGTTCTCATGTCATGG<br>CTTAGCCACCAAATACATGTAGTC	At4g34590	GGAGCAGAGGAAACGTAAACGGAT<br>CCTGAGCCGTTAGATCGTCTAGGA
At2g20570	CATCCAATGCATAACGGGACGACT<br>TGGCGGTGCTCTAAATCTCGTAGC	At4g38950	CCGTAAACTTAGTAAGGGAAGAC<br>CTTCGTTAGCTCTACATGACTC
At2g29340	CTCAATCTTATCTTGAGGACGTC<br>CCAGTAATATAAGAAGCTGCAGG	At4g39510	CCTTCCAACGCAAGTCTCCT<br>CAAACCGCTCTCATCCTTCC
At3g05600	TCTCCAAATTCGAGAAGGCA<br>GAGCTCCCAATTCAGATCCA	At5g17300	GAGCTTGAGACTCTGAAGCTGGA<br>AGACTTTGCTTCGTTGGTTCCTTG
At3g15760	TCTTGTCTATCTTCAGAGGAAACG<br>TTTCCGGTTCAATTCCCTCCT	At5g20230	CGATGTTGGTGATGATACGG<br>CGAAATCAAATTCGAGCTCGT

At3g26960	TTATAGAACCACTACGGATCAG<br>AACCTTATTTCTTGCCACAC	At5g45650	CTCAGGAACTTCAATGTCTTGTC<br>GTTAGTCATCCAAGCAGTGGTC
AtACT2	CCAGAAGGATGCATATGTTGGTGA<br>GAGGAGCCTCGGTAAGAAGA	At5g57560	AACTCCGCAGGAACAGTCAC<br>GTAAGGTTCTCCACTTGAATTCCC
AtGluTRBP	ATCTAGACTTTGTGGTTTCAGAAACAAA<br>TGGAAATGGAATCCTCACATC	AtSAND	AACTCTATGCAGCATTTGATCCACT<br>TGATTGCATATCTTTATCGCCATC
AtHEMA1	GTTGCATGTTTGTTGCTTCATAGAC<br>ATGTAACGATACTGGGACCAATACC	AtTOC159	GGGCTGTCTCTGGTAAAGTG<br>GCTCATCTTGTTGTTAAGTCCCG

Figure S1: Pigment content (A) and phenotype (B) of *Arabidopsis* wild-type Col-0 seedlings, Col-0 seedlings treated with 10 µM gabaculin (GAB) and *gun4-1* mutant seedlings 6 h after deetiolation or after 6 d growing on MS medium (control) or MS medium + 10 µM gabaculin. The contents of Chl a, Chl b and Car of the untreated controls were 0.7 ± 0.2, 0.4 ± 0.2 and 2.6 ± 0.6 ng mg fw^{-1} 6 h after deetiolation and 172 ± 20, 44 ± 3 and 57 ± 5 ng mg fw^{-1} for 6 d old seedlings respectively.

A

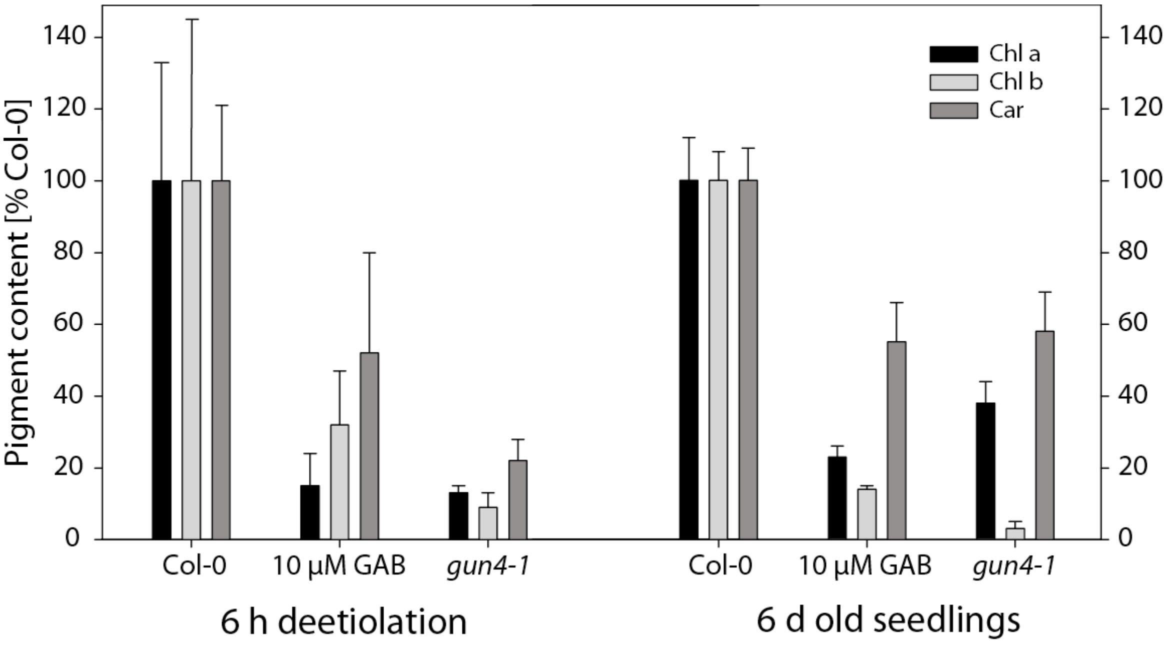

B

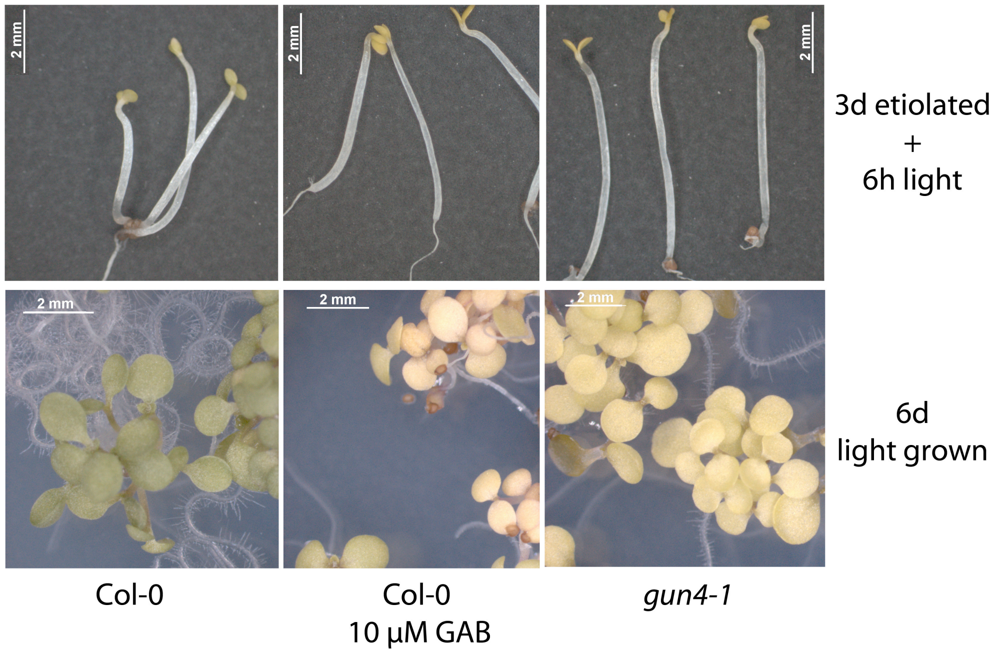

Figure S2: Pchlide content of etiolated *Arabidopsis* Col-0 seedlings grown in liquid MS-medium containing increasing concentrations of ALA. Surface sterilised seedlings were germinated and grown for 3 d in darkness under continuous shaking. Above an ALA concentration of 50 µM in the medium seedlings over-accumulate Pchlide.

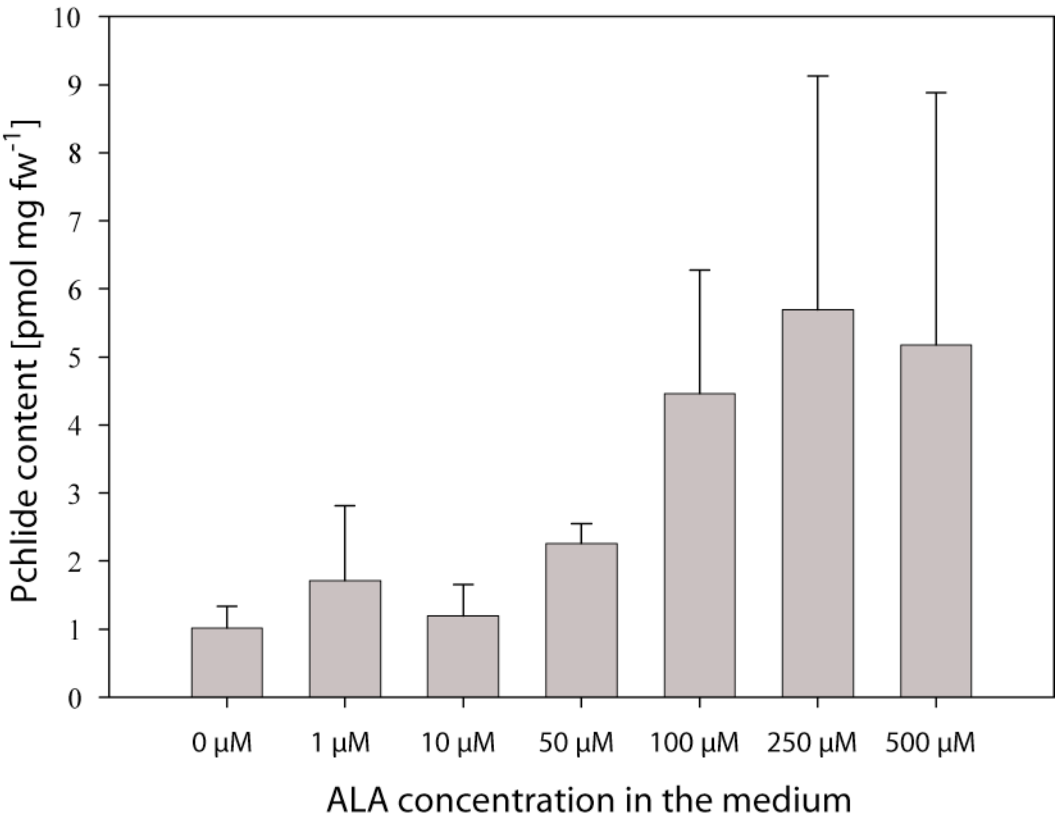

Figure S3: Phenotypes of *Arabidopsis* wild-type Col-0, *gun1-1*, and *gun4-1* treated with combinations of 1 µM norflurazon (NF), 10 µM gabaculin (GAB), and 100 µM ALA, respectively. Seedlings were grown on 0.5 MS medium supplemented with 1% (w/v) sucrose containing the indicated chemicals for 6 d under photoperiodic growth light (12 h light / 12 h dark) at 100-120 µmol photons m^{-2} s^{-1}.

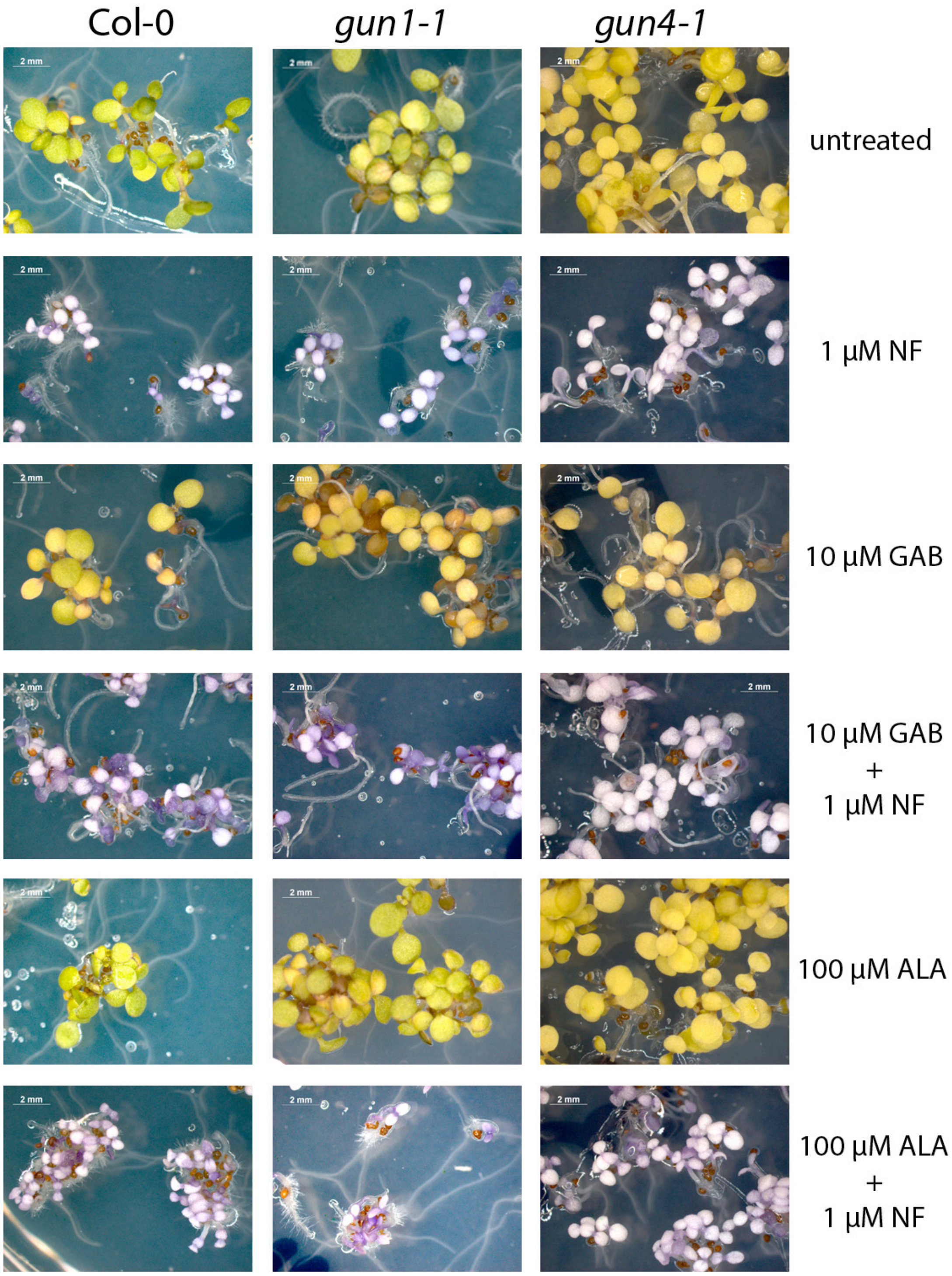

Figure S4: Pigment accumulation during deetiolation of 3 d old etiolated *Arabidopsis* Col-0 seedlings. Given are means ± S.D. of three independent extractions.

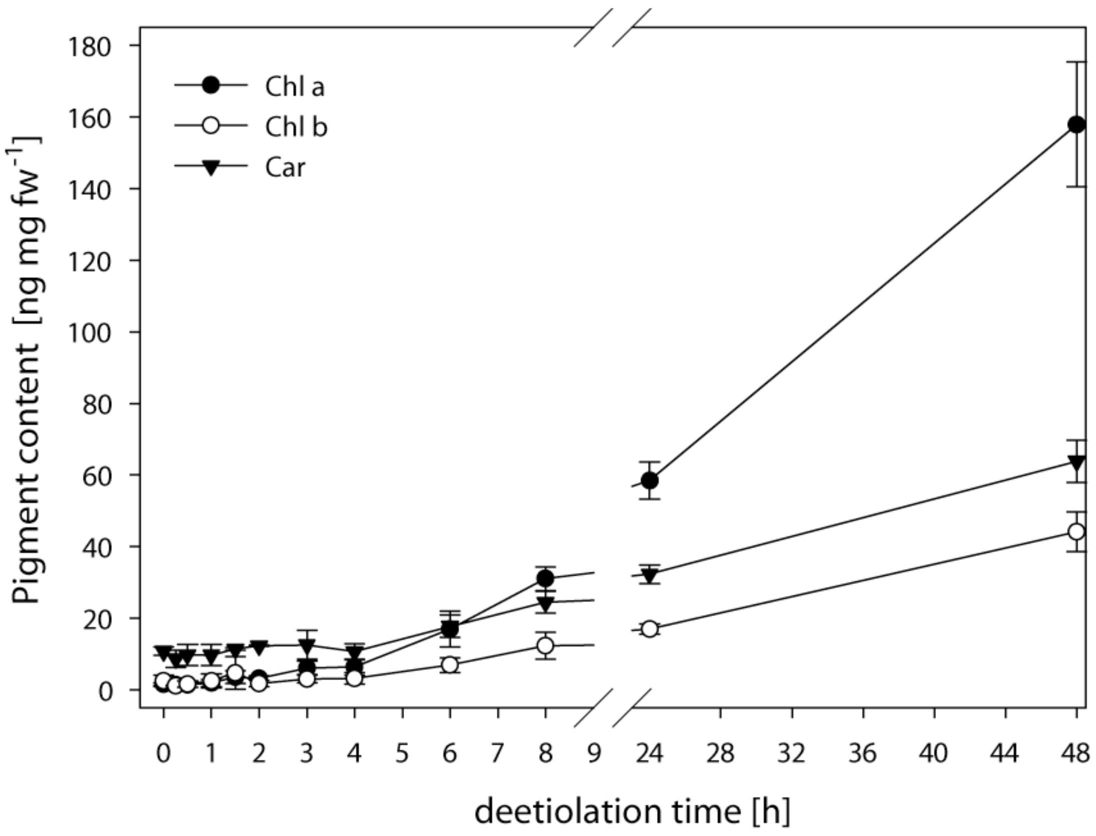

Figure S5: Over representation of gene ontology (GO) categories in genes showing decreased (A) and increased (B) transcript abundance in GAB treated *Arabidopsis* wild type Col-0 and/or *gun4-1* mutants 6 h after illumination of etiolated seedlings. All 1032 genes being downregulated (A) and 622 genes being upregulated (B) in both GAB treated and *gun4-1* seedlings, were analyzed for overrepresented GO-categories (p-value of < 0.05). Dotted lines indicate intermediate categories that are not shown. Calculation of over represented GO categories based on BiNGO 2.44.

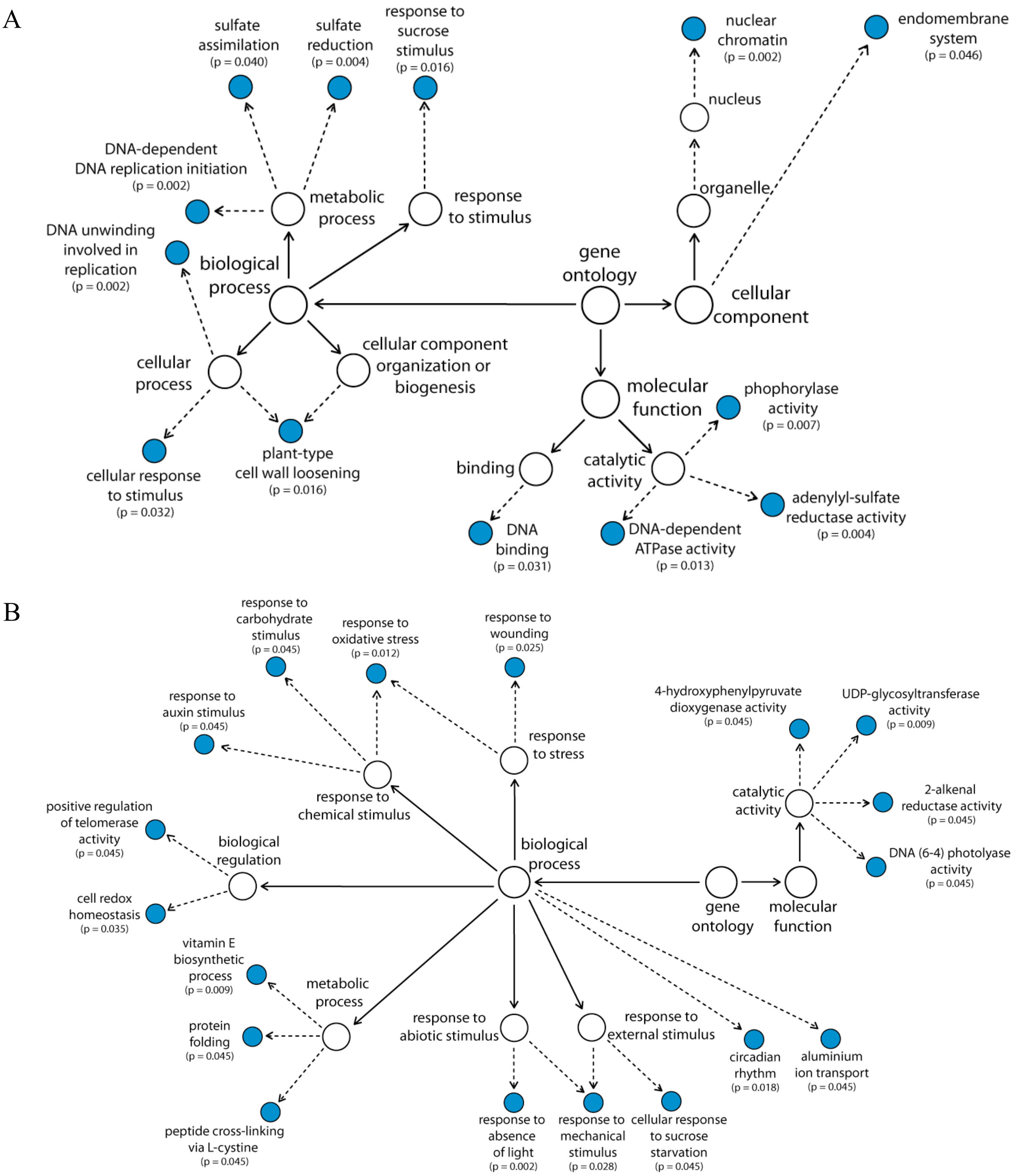

Table S2: Verification of microarray results via qRT-PCR. RNA from GAB-treated *Arabidopsis* wild type seedlings and *gun4-1* mutant seedlings was extracted 6 h and 24 h, respectively, after light exposure. Transcript abundance of a defined set of genes was determined by quantitative Real Time PCR and calculated as relative expression [$2^{-\Delta\Delta Ct}$] compared to untreated *Arabidopsis* wild type Col-0 seedlings. qRT-PCR data are given as average ± S.D. of three biological replicates. Listed are relative expressions obtained by microarray experiments (sampling 6 h after light exposure) as well as qRT-PCR in order to compare relative expressions in both experimental approaches. Relative expressions of qRT-PCR data were calculated using *AtSAND* (for 6h values) and *AtACT2* (for 24h values) as reference. Bold: Relative transcript abundance in GAB-treated or *gun4-1* seedlings (determined by qRT-PCR) significantly differs from the untreated *Arabidopsis* wild type Col-0 ($P < 0.05$).

AGI number	microarray data fold change (*P*-value)		qRT-PCR data rel. expr. ± S.D. [$2^{-\Delta\Delta_{Ct}}$] 6 h of de-etiolation		qRT-PCR data rel. expr. ± S.D. [$2^{-\Delta\Delta_{Ct}}$] 24 h of de-etiolation		Description
	GAB vs. Col-0	*gun4-1* Col-0	GAB vs. Col-0	*gun4-1* Col-0	GAB vs. Col-0	*gun4-1* Col-0	
**up-regulated genes**							
At1g05560	4.01 (0.000)	2.06 (0.000)	1.11 ± 0.03	0.80 ± 0.10	1.08 ± 0.20	0.93 ± 0.16	UDP-glucose:4-aminobenzoate acylglucosyltransferase (UGT75B1)
At1g22890	3.71 (0.000)	6.33 (0.000)	**0.67 ± 0.05**	**1.97 ± 0.47**	**2.15 ± 0.13**	1.17 ± 0.09	unknown protein
At2g19800	2.84 (0.000)	2.69 (0.000)	**1.30 ± 0.14**	0.94 ± 0.11	**1.58 ± 0.17**	**1.18 ± 0.05**	MIOX2 (Myo-inositol oxygenase 2)
At3g47340	2.68 (0.000)	5.92 (0.000)	2.85 ± 1.66	**4.30 ± 1.42**	**8.19 ± 1.41**	**11.13 ± 2.31**	ASN1 (glutamine-dependent asparagine synthase 1)
At1g05680	2.59 (0.005)	2.25 (0.003)	0.89 ± 0.33	**0.36 ± 0.09**	**1.93 ± 0.32**	0.69 ± 0.13	UDP-glucoronosyl/UDP-glucosyl transferase
At3g28740	2.46 (0.001)	2.47 (0.000)	**1.28 ± 0.05**	**1.23 ± 0.05**	1.73 ± 0.44	**2.52 ± 0.61**	CYP81D1; electron carrier/ heme binding / iron ion binding / monooxygenase/ oxygen binding
At2g17880	2.42 (0.001)	3.18 (0.000)	1.72 ± 0.70	**3.16 ± 0.83**	**2.13 ± 0.32**	**4.12 ± 0.82**	putative DNAJ heat shock protein
At4g31870	2.34 (0.002)	2.26 (0.001)	1.25 ± 0.15	1.13 ± 0.11	**1.94 ± 0.26**	0.94 ± 0.18	ATGPX7 (glutathione peroxidase 7)

At1g08630	2.31 (0.001)	8.62 (0.000)	1.16 ± 0.02	**1.55 ± 0.13**	**2.68 ± 0.18**	**4.73 ± 0.41**	THA1 (threonine aldolase 1)
At5g57560	2.22 (0.000)	6.49 (0.000)	1.21 ± 0.25	**1.98 ± 0.36**	**2.70 ± 0.52**	1.22 ± 0.53	TCH4 (touch 4); hydrolase, acting on glycosyl bonds / xyloglucan:xyloglucosyl transferase
At2g29340	2.14 (0.001)	2.04 (0.000)	1.35 ± 0.14	1.58 ± 0.26	1.70 ± 0.55	0.89 ± 0.24	short-chain dehydrogenase/reductase (SDR) family protein
At5g17300	2.07 (0.001)	2.12 (0.000)	**2.02 ± 0.38**	**2.12 ± 0.33**	**2.79 ± 0.35**	**2.96 ± 0.35**	myb family transcription factor
At5g20230	2.04 (0.005)	22.47 (0.000)	1.53 ± 0.63	1.21 ± 0.28	1.35 ± 0.30	0.84 ± 0.09	ATBCB (blue-copper binding protein)
At4g15260	2.04 (0.000)	2.06 (0.000)	0.96 ± 0.06	**0.80 ± 0.06**	1.35 ± 0.30	0.84 ± 0.07	UDP-glucoronosyl/UDP-glucosyl transferase
At3g15760	2.01 (0.001)	2.03 (0.000)	**1.29 ± 0.03**	**1.63 ± 0.17**	**2.59 ± 0.51**	**2.26 ± 0.57**	unknown protein
At3g48360	1.98 (0.001)	2.74 (0.000)	**3.33 ± 0.79**	**4.68 ± 0.94**	**6.87 ± 0.86**	**9.51 ± 1.81**	BT2 (BTB AND TAZ DOMAIN PROTEIN 2); protein binding / transcription factor/ transcription regulator
**down-regulated genes**							
At2g20570	0.30 (0.000)	0.35 (0.000)	**0.59 ± 0.08**	1.05 ± 0.22	**0.31 ± 0.02**	**0.28 ± 0.04**	GPRI1 (GBF'S Pro-rich region-interacting factor 1)
At1g61800	0.35 (0.000)	0.49 (0.000)	0.82 ± 0.08	**0.36 ± 0.04**	**0.09 ± 0.00**	**0.04 ± 0.01**	GPT2 (glucose-6-phosphate transmembrane transporter)
At3g26960	0.37 (0.000)	0.29 (0.000)	**0.69 ± 0.02**	**0.54 ± 0.05**	**0.25 ± 0.04**	**0.10 ± 0.02**	unknown protein
At4g04610	0.39 (0.019)	0.17 (0.000)	0.98 ± 0.19	**0.50 ± 0.10**	**0.24 ± 0.07**	**0.03 ± 0.00**	APR1 (APS reductase 1)
At4g28250	0.39 (0.003)	0.18 (0.000)	0.60 ± 0.23	0.82 ± 0.22	**0.43 ± 0.13**	**0.20 ± 0.03**	ATEXPB3 (Expansin B3)
At3g61820	0.41 (0.000)	0.34 (0.000)	0.87 ± 0.09	0.95 ± 0.18	**0.47 ± 0.08**	**0.16 ± 0.02**	aspartyl protease family protein
At1g56650	0.44 (0.000)	0.36 (0.000)	0.85 ± 0.12	0.78 ± 0.23	**0.14 ± 0.03**	**0.02 ± 0.00**	PAP1 (production of anthocyanin pigment 1); DNA binding / transcription factor
At5g45650	0.44 (0.001)	0.36 (0.000)	1.17 ± 0.28	1.17 ± 0.06	0.82 ± 0.15	**0.24 ± 0.06**	subtilase family protein

At1g24020	0.46 (0.017)	0.16 (0.000)	**0.83 ± 0.05**	**0.78 ± 0.06**	0.77 ± 0.10	**0.23 ± 0.03**	MLP423 (MLP-like protein 423)
At4g34590	0.47 (0.003)	0.43 (0.000)	**0.74 ± 0.03**	**0.58 ± 0.00**	**0.34 ± 0.03**	**0.15 ± 0.02**	GBF6 (G-BOX BINDING FACTOR 6)
At1g26250	0.48 (0.112)	0.13 (0.000)	**0.84 ± 0.05**	**0.57 ± 0.05**	**0.53 ± 0.08**	**0.20 ± 0.02**	putative prolin rich extensin like familiy protein
At3g05600	0.48 (0.005)	0.46 (0.001)	0.80 ± 0.13	**1.74 ± 0.07**	**0.41 ± 0.03**	**0.18 ± 0.04**	putative epoxide hydrolase
At4g39510	0.49 (0.001)	0.37 (0.000)	**0.68 ± 0.16**	**0.74 ± 0.07**	**0.43 ± 0.09**	**0.20 ± 0.02**	CYP96A12; electron carrier/ heme binding / iron ion binding / monooxygenase/ oxygen binding
At1g66940	0.49 (0.002)	0.27 (0.000)	0.87 ± 0.14	**0.64 ± 0.08**	**0.43 ± 0.05**	**0.15 ± 0.03**	protein kinase-related
At4g38950	0.50 (0.004)	0.39 (0.000)	1.03 ± 0.04	1.11 ± 0.19	**0.38 ± 0.06**	**0.25 ± 0.03**	kinesin motor family protein
**not regulated genes**							
AtHEMA1	0.98 (0.791)	0.89 (0.159)	1.11 ± 0.13	**1.27 ± 0.14**	0.84 ± 0.08	**0.70 ± 0.09**	GluTR (At1g58290)
AtGluTRBP	1.08 (0.186)	1.09 (0.134)	1.04 ± 0.06	1.08 ± 0.10	0.87 ± 0.10	0.54 ± 0.30	GluTRBP (At3g21200, PGR7)
AtTOC159	0.93 (0.352)	0.99 (0.873)	1.08 ± 0.18	**1.59 ± 0.23**	**0.75 ± 0.06**	**0.60 ± 0.12**	TOC159 (At4g02510)
AtELIP1	1.76 (1.000)	1.40 (1.000)	1.00 ± 0.09	1.43 ± 0.20	0.72 ± 0.09	**0.12 ± 0.01**	ELIP1, early light-inducible protein 1 (At3g22840)
AtELIP2	2.35 (0.607)	2.53 (0.173)	1.10 ± 0.12	1.47 ± 0.18	0.81 ± 0.12	**0.19 ± 0.03**	ELIP2, early light-inducible protein 2 (At4g14690)
AtLHCB1.2	0.94 (0.370)	0.93 (0.277)	1.06 ± 0.31	1.33 ± 0.24	0.76 ± 0.11	1.03 ± 0.08	LHCB1.2 (At1g29910)
